# Modeling clonal hematopoiesis in umbilical cord blood cells by CRISPR/Cas9

**DOI:** 10.1038/s41375-021-01469-x

**Published:** 2021-11-15

**Authors:** Friederike Christen, Raphael Hablesreiter, Kaja Hoyer, Cornelius Hennch, Antje Maluck-Böttcher, Angela Segler, Annett Madadi, Mareike Frick, Lars Bullinger, Franziska Briest, Frederik Damm

**Affiliations:** 1 Charité – Universitätsmedizin Berlin, corporate member of Freie Universität Berlin, Humboldt-Universität zu Berlin, and Berlin Institute of Health, Department of Hematology, Oncology, and Cancer Immunology, Berlin, Germany; 2 Charité – Universitätsmedizin Berlin, corporate member of Freie Universität Berlin, Humboldt-Universität zu Berlin, and Berlin Institute of Health, Department of Gynecology with Center for Oncological Surgery, Berlin, Germany; 3 Charité – Universitätsmedizin Berlin, corporate member of Freie Universität Berlin, Humboldt-Universität zu Berlin, and Berlin Institute of Health, Department of Obstetrics, Berlin, Germany; 4 German Cancer Consortium (DKTK) and German Cancer Research Center (DKFZ), Heidelberg, Germany

**Keywords:** Translational research, Myelodysplastic syndrome, Cancer genetics, Oncogenesis

## Abstract

To investigate clonal hematopoiesis associated gene mutations in vitro and to unravel the direct impact on the human stem and progenitor cell (HSPC) compartment, we targeted healthy, young hematopoietic progenitor cells, derived from umbilical cord blood samples, with CRISPR/Cas9 technology. Site-specific mutations were introduced in defined regions of *DNMT3A*, *TET2*, and *ASXL1* in CD34^+^ progenitor cells that were subsequently analyzed in short-term as well as long-term in vitro culture assays to assess self-renewal and differentiation capacities. Colony-forming unit (CFU) assays revealed enhanced self-renewal of *TET2* mutated (*TET2*^mut^) cells, whereas *ASXL1*^mut^ as well as *DNMT3A*^mut^ cells did not reveal significant changes in short-term culture. Strikingly, enhanced colony formation could be detected in long-term culture experiments in all mutants, indicating increased self-renewal capacities. While we could also demonstrate preferential clonal expansion of distinct cell clones for all mutants, the clonal composition after long-term culture revealed a mutation-specific impact on HSPCs. Thus, by using primary umbilical cord blood cells, we were able to investigate epigenetic driver mutations without confounding factors like age or a complex mutational landscape, and our findings provide evidence for a direct impact of clonal hematopoiesis-associated mutations on self-renewal and clonal composition of human stem and progenitor cells.

## Introduction

Hematopoietic stem and progenitor cells (HSPCs) are continuously generating mature blood cells. Although HSPCs divide rarely, they can accumulate somatic mutations throughout their lifespan. Mutations are acquired randomly in HSPCs with a rate of 0.07–0.86 mutations/year [1], and most of these spontaneous somatic alterations do not affect cell function. Some mutations might confer a proliferative and/or self-renewal advantage to the HSPCs, leading to preferential expansion of specific clone(s). This represents an effect mainly seen in the aging hematopoietic system. Large sequencing studies have identified recurrent mutations in blood cells of elderly individuals suffering from non-hematologic diseases [2, 3] or healthy elderly individuals [3, 4]. This phenomenon, termed clonal hematopoiesis of indeterminate potential (CHIP), is defined by the presence of a hematologic malignancy-associated gene mutation with a variant allele frequency (VAF) of at least 2% [5]. The incidence of CHIP increases in an age-related manner. Especially the epigenetic regulators *DNMT3A*, *TET2*, and *ASXL1* (DTA) have been identified to be most commonly affected in association with CHIP [2–4]. We and others have previously identified CHIP mutations in primitive Lin^−^CD34^+^CD38^−^ hematopoietic stem cells (HSCs) [6–8]. Several groups have demonstrated that similar mutations can be found in a pre-leukemic cell population in patients with full-blown acute leukemia. These clones may survive chemotherapy, persist during remission, and have a fitness advantage over normal HSCs [9–12].

To date, most knowledge about the impact of DTA mutations on the HSPC compartment was obtained from murine models. Conditional *Dnmt3a*^−/−^ models revealed that murine HSCs block differentiation in favor of self-renewal leading to a preferential expansion of *Dnmt3a*^−/−^ HSCs over wild-type cells [13]. In these conditional *Dnmt3a*^−/−^ mice, HSCs were continuously generated after serial transplantation, but they were not able to generate differentiated progenitor cells and continuously sustain hematopoiesis [14]. *Tet2* is highly expressed in multipotent and myeloid-committed progenitors isolated from mice. Disruption of *Tet2* function is accompanied by altered hematopoiesis, upregulation of HSC self-renewal and favors myeloid tumorigenesis [15–18]. In human CD34 + cells it has been demonstrated that *TET2* knockdown leads to a shift from erythroid differentiation toward granulomoncytic differentiation [19]. ASXL1 has diverse roles in histone modification and chromatin remodeling. Disruption of *Asxl1* in mice led to impaired lymphopoiesis as well as myelopoiesis, but was not sufficient to induce leukemia [20]. Other groups reported that heterozygous loss of *Asxl1* led to development of MDS/MPN-like conditions [21]. In human CD34 + cells, *ASXL1* mutations lead to impaired erythropoiesis [22]. Tothova and colleagues demonstrated that CD34 + cells from adults carrying DTA mutations expanded in a xenograft model and that distinct loss-of-function mutations were especially prominent 5 months after transplantation [23].

DTA mutations co-occur frequently and presence of multiple mutations increases the risk of hematologic cancer as compared to single mutations [24–27]. However, there is only little data from human combined knockout models, investigating additive effects of co-mutations on the phenotype of human HSPCs.

Since there is increasing evidence that CHIP mutations might occur very early in childhood, even in utero, interfere with branching, and thereby affect population dynamics of hematopoiesis [28–30], we aimed to investigate the impact of CHIP-associated mutations on young healthy human HSPCs in vitro without confounding effects of an aging microenvironment. So, we established a model to specifically introduce DTA mutations in human CD34^+^ cells from umbilical cord blood (CB). Established cell lines and mouse models as well as CD34^+^ cells isolated from adult bone marrow have a diverse genetic background, making it difficult to study the immediate effects of introduced mutations, especially if the effects of the specific mutations are subtle. In contrast, cells from umbilical CB have several advantages over adult progenitor cells due to their young age: (i) CB cells have accumulated less DNA damage, (ii) DNA damage in the stem cell compartment (e.g., environmental toxicity, nutritional stress, hypoxia) is minimized, (iii) CB cells are very stable and robust in long-term cultures. Thus, human CB cells represent an ideal cellular resource to study direct effects possibly caused by genetic modifications [23, 31, 32]. Our study therefore provides novel insights into the mechanisms of CHIP.

## Material and methods

### Sample acquisition and primary cell culture

Cord blood (CB) of 11 donors was obtained in cooperation with the Department for Obstetrics at Charité—Universitätsmedizin Berlin between March 2019 and May 2020. The institutional Ethics Committee approved the anonymous sampling of CB (EA2/234/18). The collection of umbilical CB was performed ex utero by an obstetrician or trained midwife with an umbilical CB collection kit from Macopharma (Mauvoux, France). CB samples were handled anonymously to respect the privacy of mother and child. Sample collection, handling and cell culture conditions are further detailed in Supplementary Methods (Fig. S1).

### CRISPR/Cas9-targeting via RNPs

CRISPR/Cas9-targeting of primary human stem and progenitor cells (HSPCs) was carried out via ribonucleoprotein (RNP)-based delivery as described before [33, 34]. In brief, recombinant Cas9 nuclease was complexed with synthetic guide RNAs (Table S1, Synthego, Redwood City, California, United States) in a molar ratio of 1:10. For homology directed repair (HDR)-mediated specific mutations, Cas9 and sgRNA were mixed in a 1:5 molar ratio. The HDR template [12.5 pmol] was added directly before electroporation. Electroporation was carried out using NEON transfection system with 1600 V, 10 ms, and 3 pulses. After electroporation, the cells were cultured in short-term culture medium without antibiotics, before seeding for different downstream experiments.

### Sanger sequencing, T7 endonuclease assay, and quantitative real-time PCR

DNA and RNA were isolated from cultured cells according to standard protocols. More details on PCR and real-time PCR primers, Sanger sequencing and the T7 endonuclease I (T7E1) assay are detailed in Supplementary Methods.

### SDS-PAGE and western blotting

Details are provided in Supplementary Methods (Table S2).

### Colony forming and serial replating assays

Transfected cells were resuspended in IMDM (Thermo Fisher Scientific, Waltham, Massachusetts, United States) supplemented with 2% FBS (Merck Group, Darmstadt, Germany) at a final concentration of 1 × 10^4^ cells/ml. For duplicate assays 250 µl of the cell suspension was added to 2.5 ml MethoCult^®^ H4435 medium (STEMCELL^™^ Technologies), mixed thoroughly, and 2 × 1 ml of the cell suspension was dispensed into 35 mm cell culture dishes. The cells were incubated for 12 days at 37 °C. Colonies were enumerated based on their morphology. To assess serial replating capacity, quantified cultures were resuspended in IMDM/ 2% FBS, washed twice, and viable cells were quantified. Depending on the cell concentration and viability, 1000–2500 cells were again plated in MethoCult^®^, as described above. The process was repeated up to four times, or until no colonies were detected.

### Long-term culture initiating-cell assays

The feeder cell lines Sl/Sl and M2-10B4 were obtained as a kind gift (Prof. Michael Heuser, Hannover Medical School, Germany). M2-10B4 are engineered to express the human cytokines IL-3 (interleukin-3) and G-CSF (granulocyte-colony stimulating factor) and Sl/Sl express the human cytokines IL-3 and SCF (stem cell factor). Every third passage Geneticin and Hygromycin B (both Thermo Fisher Scientific) were added to the culture medium to select transduced cells [35, 36]. The feeder cells were mixed in a 1:1 ratio and seeded into collagen coated culture vessels before irradiation with 80 Gy (rate: 2.5 Gy/min). After 24 h, the test cells were added in human long-term culture medium (HLTM: MyeloCult H5100, STEMCELL^™^ Technologies,+1 µM Hydrocortisone, Merck Group). The cells were cultured for up to nine weeks with half-medium change once a week. After long-term culture, the complete vessel was harvested, viable cells quantified via trypan blue exclusion, and the number of colony-forming cells determined via colony-forming assay in MethoCult^®^ H4435 as described above.

### Flow cytometry analysis of cell surface markers

Details are provided in Supplementary Methods (Table S3).

### Dot blot analysis of 5-methylcytosin and 5-hydroxymethylcytosin levels

The detailed protocol is provided in Supplementary Methods.

### Deep sequencing and indel analysis via CRISPRseq

DNA was extracted as described before and target sites were amplified to result in 100–200 bp fragments [37]. Further sequencing and alignment information are detailed in Supplementary Methods. The “Unknown indel analysis”-pipeline of CRISPRseq was used [23]. Aligned reads were filtered for those mapping to the DTA target amplicons. Variants were called with DeepVariant (version 0.9.0) [38] in WGS mode limited. Only insertions and deletions found at least ten times per sample were used for further analysis.

### RNA sequencing

Details are provided in Supplementary Methods.

### RIMA analysis

Details are provided in Supplementary Methods.

### Statistical analysis

Analysis of entropy was performed using the R package “entropy” [39]. Counts for each indel per sample and target were used as input for the entropy calculation. All indels that were found more than ten times were included in the analysis.

Data obtained from in vitro culture experiments was tested for assumption of normal distribution by Shapiro–Wilk or Kolmogorov–Smirnov test. Parametric tests were chosen for datasets with assumption of normality. Tests and post-tests chosen for correction of multiple testing errors (Benjamini–Hochberg, Dunn’s or Holm method) are indicated in the figure legends. All differences were considered significant with alpha = 0.05. Statistics was performed by use of GraphPad Prism 9.1.0.

## Results

### CRISPR/Cas9 modeling in primary human HSPCs affects TET2 protein function

**Fig. 1:**
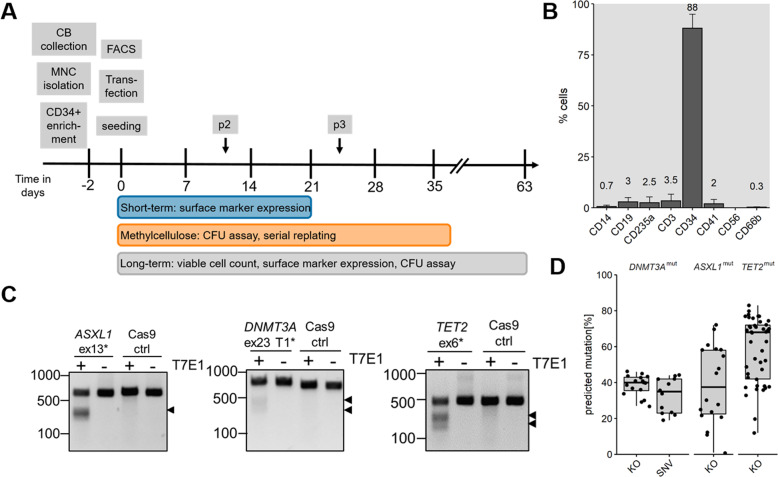
CRISPR/Cas9-mediated target-specific mutations in CD34^+^ cells from umbilical cord blood. **A** Experimental timeline from cord blood to in vitro long-term culture experiments. p2/p3 depict the time points of methylcellulose replating. FACS: flow cytometry of cell surface markers. **B** Exemplary cell surface marker distribution of CD34^+^ cells at the day of transfection. **C** T7 endonuclease I assay of each target to determine CRISPR/Cas9 efficiency. Black arrowheads highlight the digested bands. T7E1: T7 endonuclease I, ex: exon, ctrl: Cells transfected with Cas9 protein only. **D** Boxplot of predicted mutation frequencies as determined by Sanger sequencing and sequence decomposition using ICE web tool. KO: insertions and deletions. SNV: hotspot mutation R882H.

Mononuclear cells were isolated from freshly drawn umbilical CB. Subsequently, CD34^+^ cells were collected by magnetic separation and cultured for 2 days. After expansion, the cells were transfected with RNPs targeting *ASXL1* exon 13, *TET2* exon 6, or *DNMT3A* exon 23 plus the HDR templates to introduce the mutation R882H in *DNMT3A* exon 23 or a 8 bp deletion in *ASXL1* exon 13. The transfected cells were seeded for short-term culture experiments, for CFU assays, or for long-term culture assays in parallel (Fig. 1A). All in vitro culture experiments were carried out with bulk cells.

Before transfection, the enriched CD34^+^ cells were checked for surface marker expression (Fig. 1B, Fig. S2) and at least 80% CD34^+^ enrichment was obtained for 9 out of 10 CB samples. Seven days after transfection the editing efficiency was estimated via either T7 endonuclease assay (Fig. 1C) or Sanger sequencing and sequence decomposition [40]. Sanger sequencing was repeated after 14 and 21 days of culture. We obtained a mean mutation efficiency of 38%, 39%, and 58% for *DNMT3A*, *ASXL1*, and *TET2*, respectively. The 8 bp deletion in exon 13 of *ASXL1* was included in the indel frequency. The point mutation in *DNMT3A* exon 23 was introduced in 33% of cells (Fig. 1D). The high mutation rate obtained via CRISPR-modeling facilitated the work using bulk cell cultures, which provides more insights in dynamics that involve wild-type (WT) cells. Mutations and functional knockouts were analyzed on mRNA and protein level for all targets without clear evidence of mutant mRNA or protein decay (Fig. S3). In addition, a downregulation of 5-hydroxymethylcytosin conversion for *TET2*^mut^ cells could be confirmed (Fig. S4).

### TET2^mut^ cells show altered differentiation-associated expression pattern and serial replating capacity in liquid culture

**Fig. 2:**
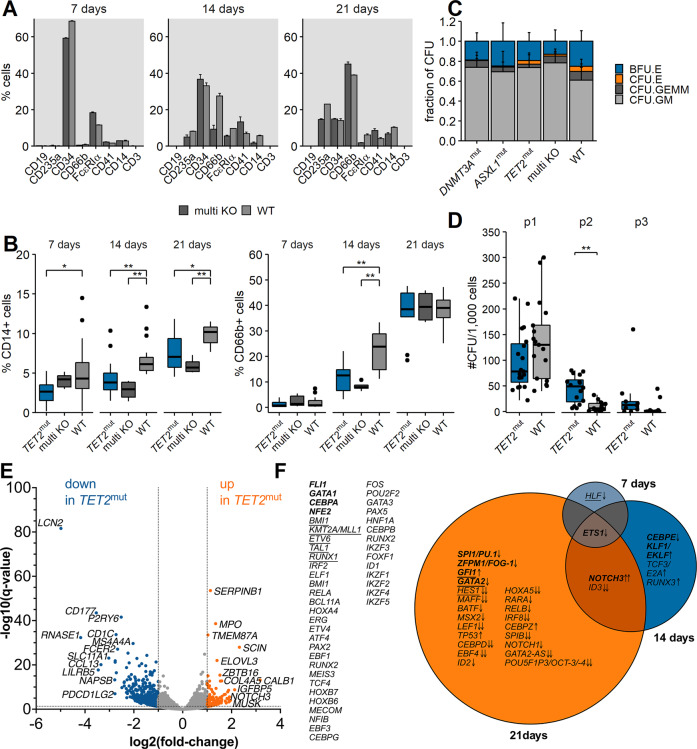
Differentiation and self-renewal capacities of mutated CD34^+^ cells. **A** Cell surface marker expression of multi KO vs. WT cells 7, 14, and 21 days after transfection. One exemplary biological sample is shown. Frequencies were normalized to 100% for all depicted cell populations. Bars and error bars show mean (SD) of two transfection replicates. **B** CD14 (left) and CD66b (right) surface marker expression of *TET2*^mut^, multi KO, and wild-type cells 7, 14, and 21 days after transfection. Boxplots show all biological and technical replicates per condition. *p < 0.05, **p < 0.01, Wilcoxon rank-sum test, corrected for multiple testing. **C** Relative distribution of CFU types for all biological and technical replicates after the first seeding. BFU.E: burst-forming unit erythrocyte, CFU.E: colony-forming unit erythrocyte, CFU.GEMM: colony-forming unit granulocyte, erythrocyte, macrophage, megakaryocyte, CFU.GM: colony-forming unit granulocyte, macrophage. **D** Serial replating assay of *TET2*^mut^ vs. WT cells. Boxplots show all technical and biological replicates. The number of passages is indicated above the plot. **p < 0.01, Wilcoxon rank-sum test. **E** Differential Expression of genes in *TET2* mutant cells. Global transcriptome of *TET2*^mut^ cells vs. wild-type counterparts was determined by RNA Sequencing of four replicates derived from two biological samples 7, 14, and 21 days after transfection. Volcano blot shows differentially expressed (DE) genes after 21 days of both samples pooled in one analysis (Table S4). Log2-fold change is shown on the *x*-axis and the *y*-axis shows the corresponding −log10 of the adjusted *p* values. **F** Longitudinal changes of gene expression regulators after *TET2* perturbation. Significantly DE genes (*q* < 0.05 either in the pooled analyses or in both of the samples in the same direction) with a minimum log2-fold-change of ±0.4 were matched with a predefined list of transcriptional regulators of hematopoiesis derived from the literature (Table S4). VENN diagram shows (common) transcriptional regulators differentially expressed at the different times points. Arrows indicate up- or downregulation, double arrows indicate log2-fold-change >1/<−1. Transcriptional regulators associated with myeloid differentiation are highlighted in bold, underlined genes are associated with stem cell properties.

By culturing the cells in liquid culture medium for 3 weeks, immediate effects of introduced mutations on cell surface markers and global gene expression were determined. After 7, 14, and 21 days, cell surface marker expression of the bulk cells were analyzed via flow cytometry. We first examined cells transfected with Cas9-RNP targeting all three genes simultaneously (multi KO) to identify short-term effects. We observed a difference in CD66b- and CD14-marker expression between the multi KO and the wild-type cells, whereas for the other surface marker no pattern was detectable (Fig. 2A and Fig. S5A). To dissect these immediate effects further, seven biological replicates targeted with *TET2*-RNP (*TET2*^mut^) and four samples targeted with *ASXL1*- or *DNMT3A*-RNPs (*ASXL1*^mut^ and *DNMT3A*^mut^, respectively) were analyzed in total. For *TET2*^mut^, a significantly reduced CD66b-marker expression was observed after 14 days of culture, which was not detected anymore after 21 days of culture. CD14-marker expression was significantly reduced in *TET2*^mut^ for all time points (Fig. 2B, *p* < 0.05). We did not find a distinct expression pattern for *ASXL1*^mut^ or *DNMT3A*^mut^ cells (Fig. S5B). Thus, in our model only the introduction of *TET2* mutations led to a delay in mature myeloid surface marker expression in short-term liquid culture.

To investigate whether the introduced mutations affect committed progenitor maturation, we performed colony forming and replating assays. We did not observe significant differences in the relative distribution of CFU types after the initial plating (Fig. 2C), suggesting an equal distribution of different progenitor types for all conditions. To assess the self-renewal capacity of the progenitor cells, we performed a serial replating assay. An enhanced serial replating capacity was only observed for *TET2*^mut^ cells (Fig. 2D, *p* < 0.01). This observation suggested that committed progenitors carrying a *TET2* mutation gained enhanced self-renewal capacities. However, exhaustion was observed after three replating series (Fig. S6). Neither *DNMT3A*^mut^ nor *ASXL1*^mut^ led to a comparable result (Fig. S7A).

In order to investigate perturbation of hematopoietic lineage commitment, we performed RNA sequencing. Gene expression analysis 7, 14, and 21 days after knockout of *TET2* revealed increasing alterations in the gene expression patterns over time, with a total number of 84, 442, and 2256 differentially expressed genes after 7, 14, and 21 days, respectively (log2-FC < −0.4 or >0.4; *q* < 0.05). Thereby, *TET2* knockout affected a large number of genes associated with the regulation of hematopoietic differentiation, such as an early downregulation of *CSF2RA*. We found a common downregulation of *ETS1* after 7 days, which persisted after 14 and 21 days. Later events were the upregulation of *NOTCH3* and the downregulation of *ID3*. After 21 days, a large number of myeloid transcription factors, such as *PU.1*, *FOG-1*, *GFI1*, or *GATA2*, and myeloid lineage associated genes were found perturbated in relation to *TET2* wild-type cells (Fig. 2E, F, Fig. S8, Table S4).

In summary, these results indicate that *TET2* mutations have an effect in terms of increased self-renewal but delayed differentiation capacities of hematopoietic progenitor cells, especially affecting the myeloid lineage, even in short-term culture conditions and without further aging-associated factors.

### DTA-mutations affect self-renewal capacities of HSPCs

**Fig. 3:**
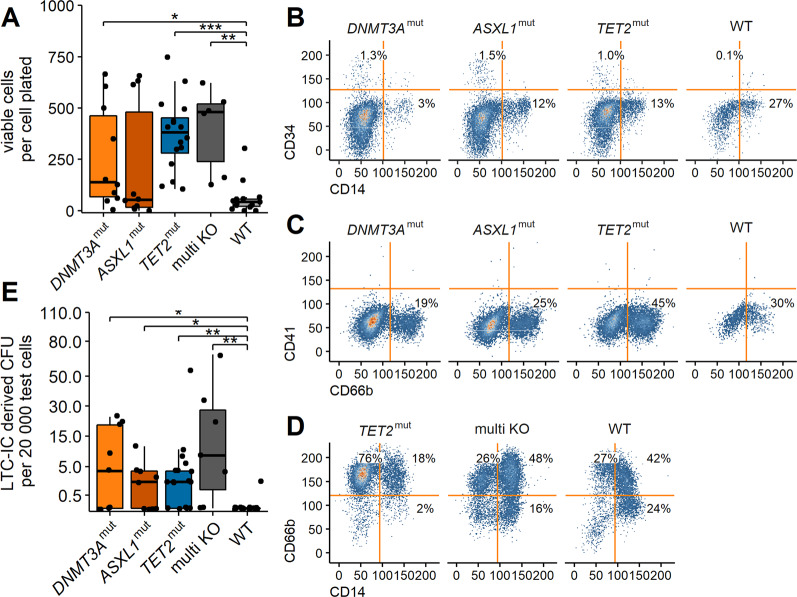
Self-renewal capacities of mutated HSPCs after long-term culture. **A** Transfected cells were cultured for up to 9 weeks on irradiated feeder cells. The number of viable cells was determined via trypan blue exclusion and normalized to the number of cells initially plated. **B**–**D** Flow cytometry analysis of viable cells after long-term culture. One representative replicate of one biological sample is shown. **E** Long-term culture initiating cell derived CFU assay. The number of LTC-IC derived CFU was normalized to the number of seeded cells. The *y*-axis was square root transformed for better visualization. *p < 0.05, **p < 0.01, ***p < 0.001 Wilcoxon rank-sum test corrected for multiple testing.

Primitive progenitors can be investigated in long-term culture initiating-cell assays [35]. The cells were cultured for up to nine weeks on a feeder layer. After harvesting, the viable cell count was determined for each replicate. Viable cell numbers in *DNMT3A*^mut^ and *ASXL1*^mut^ samples differed strongly between donors. In *TET2*^mut^ and multi KO samples the variability was less pronounced. For all mutant samples, except *ASXL1*^mut^, a significantly increased number of viable cells compared to the wild type sample was detected (Fig. 3A, *p* < 0.05). Thus, we showed the presence of primitive progenitors and/or committed progenitors supporting continuous production of mature myeloid cells in the samples. These cells had enhanced self-renewal capacities compared to the wild-type population. Flow cytometry analysis of the viable cells confirmed that CD34^+^ progenitors were present after long-term culture. In trend, the frequency of CD34^+^ cells was higher in the mutants compared to the wild-type samples (*DNMT3A*^mut^: 1.31 ± 0.4%, *ASXL1*^mut^: 2.71 ± 1.77%, *TET2*^mut^: 0.52 ± 0.38%, WT: 0.10 ± 0.12%, Fig. 3B). After long-term culture, the majority of cells expressed myeloid surface markers (Fig. 3C, D), irrespective of the DTA genotype, which was expected since especially myelopoiesis is supported in the applied culture condition.

Finally, to determine the number of primitive progenitors (also termed long-term culture-initiating cells, LTC-IC) that were present in the samples, a CFU assay was performed after long-term culture. LTC-IC derived CFU were detected in all mutant samples (Fig. 3E, *p* < 0.05). Interestingly, 71% of *TET2*^mut^ (*n* = 17) as well as multi KO (*n* = 7) samples were able to generate colonies. The mean number of colonies per 20.000 test cells was 8.3 and 27.2 for positive *TET2*^mut^ and multi KO samples, respectively. Of the 9 investigated *DNMT3A*^mut^ and *ASXL1*^mut^ samples, we detected colonies in 5 (56%) with a mean number of 15.8 and 4.8 colonies per 20.000 test cells for positive *DNMT3A*^mut^ and *ASXL1*^mut^ samples, respectively. Collectively, these data indicate that the introduced mutations enhance the self-renewal capacities of HSPCs. The differences in colony forming efficiency and colony numbers indicate that the mutations effect the target cells in a different manner and that self-renewal capacities are further enhanced by the presence of multiple mutations.

### Mutated cell clones expand preferentially in long term culture

**Fig. 4:**
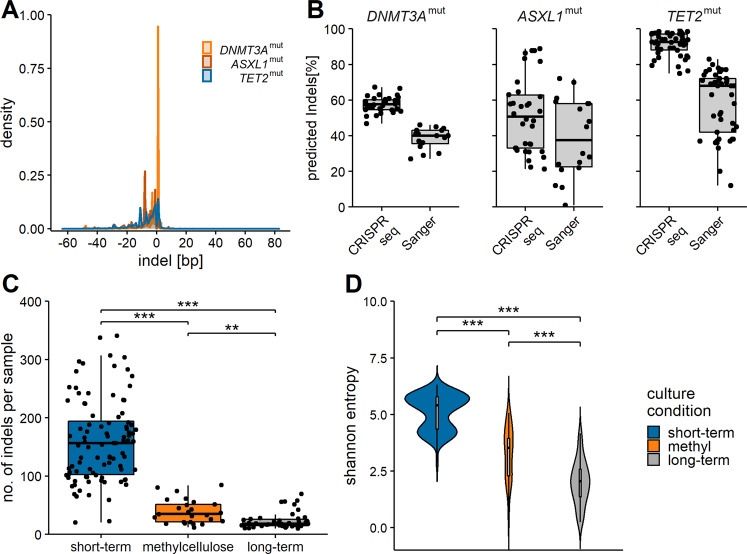
Indel composition of CRISPR-targeted HSPCs. **A** Width in bp as determined by CRISPRseq analysis of targeted deep sequencing data. **B** Comparison of predicted indel frequencies per sample via CRISPRseq vs. Sanger sequencing and indel decomposition. **C** Number of indels per sample as determined by CRISPRseq analysis. The samples are categorized by culture condition, independent of target and time point. **D** Shannon entropy analysis as measure of diversity of found indels. Samples are categorized by culture condition, independent of target and time point. **p < 0.01, ***p < 0.001 Wilcoxon rank-sum test corrected for multiple testing.

To investigate whether distinct clones preferentially expanded during the long-term culture period, a targeted deep sequencing was performed to determine the indel spectrum within one sample. Further, distinct indel types were tracked serially over various time points to analyze clonal behavior within a sample. Target amplicons were sequenced with a mean sequencing depth of ~24.000× followed by CRISPRseq mutational analysis [23] (Fig. 4A and Table S5). Comparison of mutation frequencies obtained via deep sequencing with Sanger sequencing revealed that the frequencies were underestimated by Sanger sequencing (Fig. 4B). Interestingly, short-term culture samples showed a higher number of distinct indel types with decreasing numbers of distinct indels in samples from methylcellulose and long-term cultures (Fig. 4C). The overall mutation frequency did not change between the different time points of sampling (Fig. S9). This indicated, that especially in the selective culture conditions (methylcellulose and long-term culture) clonal expansion took place. Next, Shannon entropy analysis was performed to measure clonal diversity within the samples. Samples from short-term cultures had the highest entropy, meaning that the indel composition was highly diverse in these samples. The entropy decreased for methylcellulose and long-term culture samples (Fig. 4D). With these results, we could demonstrate that distinct clones were preferentially expanding in the selective culture conditions. In order to address the question, whether non-randomly induced indels, as outcome of genome editing induced by classical microhomology-mediated end joining (c-MMEJ), show an intrinsic selective advantage on our in vitro experiments, we performed a Rational Indel Meta-Analysis (RIMA) [41]. C-MMEJ-associated microhomology was found in 28% of all deletions, with a range of 15.8% in 1–5 bp deletion to 58.5% in 11–20 bp deletions. We also found a considerable increase of VAF for c-MMEJ-associated deletions for all three DTA genotypes. However, the relative increase of clone size of MMEJ-associated mutations did not differ from that of clones of non-MMEJ offspring, suggesting that the observed bulk phenotype is rather a result of more than one specific mutagenic mechanism. (Fig. S10).

**Fig. 5:**
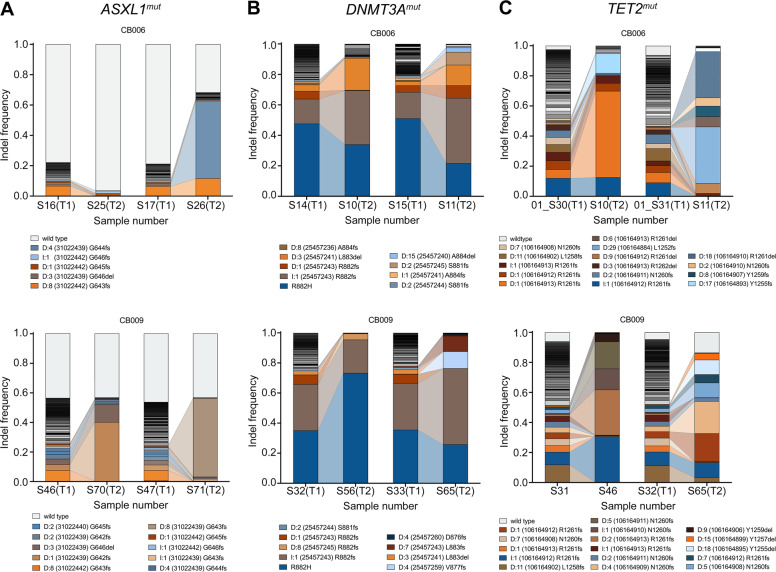
Expansion of distinct cell clones in long-term culture. Representative examples of two replicates of two independent cord blood samples (CB006 and CB009) after 7d and long-term culture. Distribution of mutations in (**A**) *ASXL1*^mut^, (**B**) *DNMT3A*^mut^, and (**C**) *TET2*^mut^ are shown before (T1) and after (T2) long-term culture. Different colors represent different mutations. Only the most prominent mutations are labelled. In Figs. S11, S12, S13 all replicates are shown.

We further analyzed which types of mutations were primarily found in expanding clones. To this end, 14 replicates from long-term cultures were investigated for each mutation target. In 50% of the investigated *ASXL1*^mut^ long-term culture samples the mutation frequency decreased compared to short-term culture samples (Fig. S11A vs. S11B). Nevertheless, we observed distinct indel types that were particularly prominent after long-term culture (Fig. 5A and S11B) such as frameshift mutations at the amino acid positions G642, G643, and G644 in the long-term culture samples.

In *DNMT3A*^mut^ long-term culture samples, two mutations were predominantly found (Fig. 5B): the point mutation R882H and a 1 bp insertion at position 25457243. The latter resulted in a frameshift at codon R882 with a frameshift elongation of the protein. In general, we observed a decrease in the overall mutation frequency in one sample (Fig. S12). These data showed that wild-type cells were not overgrowing mutated cell clones. In 9 out of 14 long-term culture samples the 1 bp insertion expanded while the point mutation either remained stable or was overgrown. The point mutation overgrew other indels in only two investigated samples (Fig. S11B). Distinct mutations seemed to enhance the expansion capacities of cell clones and especially distinct indel types conferred a proliferative advantage to the cells.

*TET2*^mut^ samples generally showed a stable overall mutation frequency with few indels expanding over the long-term culture period (Fig. 5C and Fig. S13). Thus, the indel composition was much more diverse in *TET2*^mut^ long-term culture samples compared to *ASXL1* and *DNMT3A* mutations.

## Discussion

In this study, we investigated the impact of clonal hematopoiesis-associated gene mutations on healthy HSPC development. We used cells from umbilical CB to investigate the cell-intrinsic effects of DTA mutations in short-term and long-term analyses without confounding factors linked to an aging system.

For both murine [42] and human [19] in vitro cultures, impaired myeloid differentiation has been described for *TET2* mutant and knockout cells. We could confirm that differentiation marker expression of monocyte and granulocyte markers was delayed in *TET2*^mut^ cells. We further demonstrated a perturbated myeloid expression phenotype in *TET2* altered samples, an effect that has been described previously [43]. The observed alterations of myeloid transcription factor expression (e.g., *GATA2, SPI1/PU.1, GFI1, KLF1*) might contribute to this effect indirectly, as well as altered accessibility to their target motifs due to changes in the DNA methylome by TET2 impairment itself [44]. Furthermore, alterations associated with leukemogenesis, such as perturbation of Notch Signaling [45, 46] or repression of SPI1/PU.1 and IRF8 transcription factors [47], were detected on a transcriptional level, emphasizing a role of CHIP mutations in later transforming events.

With the model established here, we were able to demonstrate enhanced self-renewal capacities for *TET2*^mut^ committed progenitors as determined via short-term serial replating assays. This is in line with previous reports from murine knockout models [15, 17, 48]. Enhanced serial replating capacities were also reported for *Dnmt3a* knockout models in mice [49], which we could not recapitulate in our experimental design. Our data indicate that introduced *DNMT3A*^mut^ and *ASXL1*^mut^ do not have as severe effects on the stem and progenitor cell compartment in vitro as complete or conditional knockouts obtained from in vivo models. Here, the lack of respective microenvironmental niches might interfere with full phenotypic consequences.

However, in long-term cultures effects on self-renewal capacity of the HSPC compartment could be demonstrated for all DTA mutations. It has been reported that human cells carrying clonal hematopoiesis-associated mutations are indeed able to support long-term hematopoiesis as well as clonal expansion in transplanted mice [23].

We were able to observe increased colony-forming capacities in all DTA-mutated CD34^+^ clones, with the strongest skewing in multi KO samples, indicating a cooperative manner of mutations. These findings are in line with published data that have shown an increasing risk for hematologic cancer development and multiple CHIP mutations [26].

We observed that especially *DNMT3A*^mut^ and *TET2*^mut^ cells are able to support long-term myelopoiesis in vitro, leading to the assumption that long-term HSCs in these conditions might have enhanced self-renewal. *ASXL1*^mut^ cells did not show increased cell production after long-term culture, nevertheless the number of LTC-IC derived CFUs was significantly increased for all mutants compared to the control group. It therefore seems that a proportion of mutated HSPCs gained enhanced self-renewal capacities. We additionally found viable CD34^+^ cells in the mutated long-term culture samples, indicating that these have indeed self-renewed over the long-culture period of up to nine weeks even in in vitro settings.

Interestingly, in *ASXL1*^mut^ and *DNMT3A*^mut^ samples, the viable cell population after long-term culture was predominantly formed from a small number of distinct cell clones. In *ASXL1*^mut^ samples, we found frameshift mutations at amino acids G642 to G644 to be enriched. Frameshift mutations at these positions have been reported frequently and are associated with a dismal prognosis [6, 50–52]. In *DNMT3A*^mut^ samples, we observed a high frequency of the specifically introduced R882H mutation, a well-known hotspot mutation in AML and to lesser extent in CHIP [6, 53]. However, this mutation did not lead to preferential expansion in the majority of samples. In contrast, *TET2*^mut^ samples from long-term culture clones revealed a more heterogeneous clonal composition within a sample as well as between samples. Therefore, our data suggest that different cell populations profit from the introduced mutations in a gene-specific fashion. Based on sorted cell populations from individuals with clonal hematopoiesis, we and others previously have provided evidence that *DNMT3A* mutations occur in very early HSCs whereas *TET2* mutations favor especially myeloid-committed progenitors [6, 8].

The strength of our model is the use of healthy, young cells and tracking the mutation frequency and expansion over the period of up to 9 weeks, which permitted the preferential expansion of either primitive or more committed progenitors independently of confounding factors such as aging processes or pre-existing somatic mutations. We were therefore able to demonstrate that DTA mutations directly influence the behavior of human HSPCs and confer enhanced self-renewal capacities to the cells, independent of the physiological context. Our model thereby provides a novel experimental setup, which offers functional information about genetic determinants of early differentiation processes in human HSPCs without the bias of already acquired somatic mutations. It can now be applied for long-term in vitro studies further exploring the role of DTA-mutations in response to genotoxic agents. This application can help to better understand clonal selection and the development of secondary hematopoietic malignancies following anti-cancer treatment in patients with clonal hematopoiesis.

## Supplementary information

Christen et al_Supplementary Information

Christen et al_Supplementary Table S4

Christen et al_Supplementary Table S5
