# Supplementary material for: Modeling clonal hematopoiesis in umbilical cord blood cells by CRISPR/Cas9
Source: Leukemia. 2021 Nov 15;36(4):1102–10. doi: 10.1038/s41375-021-01469-x (PMC8979818; doi:10.1038/s41375-021-01469-x)
Supplement: Supplementary file 1 — Christen et al_Supplementary Information [file 41375_2021_1469_MOESM1_ESM.pdf]

Supplementary Information

Modeling clonal hematopoiesis in umbilical cord blood cells by CRISPR/Cas9

Friederike Christen[1], Raphael Hablesreiter[1], Kaja Hoyer[1], Cornelius Hennch[1], Antje Maluck-Böttcher[1], Angela Segler[2,3], Annett Madadi[3], Mareike Frick[1,4], Lars Bullinger[1,4], Franziska Briest[1,§], and Frederik Damm[1,4,§,*]

Supplementary methods

Sample Acquisition and Primary Cell Culture

The freshly collected blood was filtered through a 40-µM strainer and diluted with one volume PBS/ 1 mM EDTA. Mononuclear cells were isolated via density centrifugation. After washing the cell pellet, remaining erythrocytes were eliminated by incubating the cell pellet for 30 min at 4 °C in red cell lysis buffer (155 mM NH_4Cl, 10 mM $KHCO_3$, 0.1 mM EDTA, pH 7.4). Viable cells were quantified via trypan blue exclusion in a Neubauer chamber. Stem and progenitor cells were enriched by magnetic separation using MACS CD34 MicroBead Kit UltraPure (Miltenyi Biotec, Bergisch Gladbach, Germany) according to the manufacturer's instructions. Purified cells were cultured in StemSpan SFEM II (STEMCELL™ Technologies, Vancouver Canada) expansion medium supplemented with SCF, FLT3-L, and TPO[1] (100 ng/ml each, all from PeproTech, Inc., Rocky Hill, New Jersey, United States).
For differentiation analysis, the primary CB derived cells were cultured in StemSpan SFEM II supplemented with SCF, FLT3-L (50 ng/ml each), TPO, IL-6 (20 ng/ml each), and IL-3 (10 ng/ml) for up to 3 weeks (short-term culture medium). In Figure S1 different medium compositions are shown. Unmodified cells were cultured in different cytokine combinations to determine best conditions for expansion and short-term culture. For CD34+ expansion after transfection, we used medium B. For short-term culture experiments, the cells were cultured in medium D. The purpose of our short-term liquid culture system was to facilitate unbiased differentiation because we wanted to analyze the direct consequences of DTA mutations in an unconfounded environment.

Sanger Sequencing, T7 Endonuclease assay, and quantitative real-time PCR

DNA was isolated from cultured cells using DNA Mini Kit according to the manufacturer's instructions (Qiagen, Hilden, Germany). Polymerase chain reaction (PCR) was carried out using HotStarTaq DNA polymerase (Qiagen). The following primers were used for target specific amplification: *ASXL1* 5'-tgtaaaacgacggccagtGGTCAGATCACCCAGTCAGTT-3' and 5'-caggaaacagctatgaccAGCCCATCTGTGAGTCCAAC-3'; *DNMT3A* 5'-GCACATGGTTGGTGATCTGA-3' and 5'-GGAAGGGAGCTTGGTTTTGT-3'; *TET2* 5'-tgtaaaacgacggccagtGCTGCAAGTGACCCTTGTTT-3' and 5'-caggaaacagctatgaccCTTATCGCATGACTGCCAAA-3'. The M13-tag was added to the primer sequences to facilitate Sanger sequencing. The PCR products were purified using AMPure Beads XP (Beckmann Coulter, Brea, California, United States) or PCR purification Kit (Qiagen) according to the manufacturer's instructions. The purified PCR products were normalized to 2-10 ng/µl and analyzed by Sanger sequencing. The obtained raw data were analyzed for introduced mutations via the web based application ICE (Inference of CRISPR indels, Synthego[2]).
For T7 endonuclease I (T7E1) assay, 5 µl of the purified PCR products were mixed with 2 µl NEB buffer 2 (New England Biolabs®, Ipswich, Massachusetts, United States) , denatured for 10 min at 95 °C. Hybridization was carried out by ramping down to 85°C by 2 °C/s and ramping down to 25 °C by 0.1 °C/s. After cooling down to 4 °C, 10 µl of the reaction was transferred into a new tube and mixed with 2 U T7 endonuclease I (New England Biolabs®). The reaction was incubated for 15 min at 37 °C and stopped by adding 1 µl 0.25 M EDTA. The reaction was loaded on a 1.5% agarose gel.

RNA from cultured cells was isolated using RNeasy Mini Kit (Qiagen) according to the manufacturer's instructions. cDNA was synthesized using M-MLV reverse transcriptase and random primers (both Invitrogen, Carlsbad, California, United States). Quantitative real-time PCR (qRT-PCR) was carried out using Luna® Universal qPCR Master Mix (New England Biolabs®) with the following primers: *ASXL1* 5'-GGAGAAGGATGAAGGACAAACA-3' and 5'-GTCATTGGAGCATCCGAGTAG-3'; *DNMT3A* 5'-GCCCATTCGATCTGGTGATT-3' and 5'-GGCGGTAGAACTCAAAGAAGAG-3'; *TET2* 5'-AGGTTTGGACAGAAGGGTAAAG-3' and 5'-CGAACCACCCACTTAGCAATA-3'. *GAPDH* was used as endogenous control (5'-GAAGGTGAAGGTCGGAGTCA-3' and 5'-AATGAAGGGGTCATTGAT-3').

SDS-PAGE and Western Blotting

Cells were lysed in protein lysis buffer (50 mM Tris, 150 mM NaCl, 1 mM EDTA, 1% NP-40) using a bioruptor sonificator (15 cycles, 15 s on/ 15 s off). The protein lysates were mixed with 4x Laemmli buffer (Bio-Rad Laboratories, Hercules, California, United States) containing 10% β-mercaptoethanol and proteins were separated by electrophoresis using 8-12% SDS-PAGE gels in a Mini-PROTEAN® Tetra Cell system (Bio-Rad Laboratories). Subsequently, proteins were transferred onto Amersham™ Protran® Premium Western-Blotting nitrocellulose membrane (pore size 0.45 µm, Cytiva, Marlborough, Massachusetts, Untied States). Immunodetection was carried out using antibodies listed in Table S2 according to the manufacturer's instructions. Protein bands were visualized using ECL™ Prime Western Blot reagent (Cytiva) and ImageQuant LAS 4000 system (GE Healthcare, Chicago, Illinois, United States). Band intensities were quantified using ImageJ 1.48v. Intensities were normalized to β-Actin and calculated relative to the control sample.

Flow Cytometry Analysis of Cell Surface Markers

Cells were harvested and washed twice in PBS/ 0.5% BSA. The cell pellets were resuspended in antibody mix in PBS/BSA (Table S3). Cells were analyzed on BD FACS™ Canto II flow cytometer (BD: Beckton, Dickson and Company, Franklin Lakes, New Jersey, United States). Analysis and gating was performed in FlowJo v.10 (BD).

Dot Blot Analysis of 5-Methylcytosin and 5-Hydroxymethylcytosin levels

DNA was extracted by isopropanol precipitation and the concentration of all samples was adjusted. DNA was denatured for 5 min at 99 °C and spotted on a positively charged nylon membrane (Merck Group) in 2-fold serial dilutions. The membrane was air-dried for 30 min, UV cross-linked for 3 min on a transilluminator (350 nm), and blocked in 5% milk powder in TBS-T at 4 °C over night. After washing the membrane three times, primary antibodies against 5-hydroxymethylcytosin (Active Motif, Inc., Carlsbad, California, United States) and 5-methylcytosin (Cell Signaling Technology, Danvers, Massachusetts, United States) were added in 5% BSA in TBS-T over night at 4 °C (Table S2). The membranes were again washed three times in TBS-T and incubated with secondary antibodies for 90 min. Chemiluminescence was detected using the ImageQuant LAS 4000 system. As loading control, total DNA was stained with 0.2% methylene blue in 0.3% sodium acetate for 15 min. Signal intensities were

determined using ImageJ 1.48v. All Dot Blot signals were normalized to the methylene blue loading controls.

Deep Sequencing and Indel Analysis via CRISPRseq

Deep sequencing was performed for advanced indel detection. Following primers were used: *ASXL1* 5'-GGACCCTCGCAGACATTAAA-3' and 5'-CTCACCACCATCACCACTG-3'; *DNMT3A* 5'-CTTCAGCGGAGCGAAGAG-3' and 5'-GGTCCTGCTGTGTGGTTAG-3'; *TET2* 5'-CTGTGAGGCTGCAGTGATT-3' and 5'-CAACCAAAGATTGGGCTTTCC-3'. The resulting amplicons were pooled without overlap and indexing for Illumina sequencing was performed using NEBNext Ultra DNA Library Prep Kit and NEB Next Multiplex Oligos for Illumina (New England Biolabs®) according to the manufacturer's instructions. The libraries were single-end sequenced on a MiSeq sequencer using a MiSeq reagent Kit v2 (300 cycles, Illumina, San Diego, California, United States). Sequencing reads were aligned to hg19[3] using BWA-MEM[4]. The "Unknown indel analysis"-pipeline of CRISPRseq was used as described by Tothova et al.[5]. Aligned reads were filtered for those mapping to the target amplicons of *ASXL1*, *DNMT3A*, and *TET2*. Single nucleotide variants were called with DeepVariant (version 0.9.0)[6] in WGS mode limited to the gene of interest. Mutated reads were uniquely labelled as follows: D for deletion, I for insertion; size of the indel; start position (*i.e*. I:1 (25457243)). Only insertions and deletions found at least 10 times per sample were used for further analysis.

RIMA Analysis

Rima was used in batch analysis mode according to the guidelines previously published[7] for indel analysis (sgRNAs: TET2: ACGGCACGCTCACCAATCGC;
DNMT3A: CGTCTCCAACATGAGCCGCT; ASXL1: GCCACCACTGCCATCGGAGG).

RNA Sequencing

Cells were harvested on different time points and snap frozen until use. For mRNA-Seq double-indexed libraries were prepared with TruSeq™ Stranded mRNA Library Prep Kit (Illumina, #20020595). Libraries sizes distribution was assessed using Agilent TapeStation. Libraries were sequenced on Illumina NovaSeq 6000 SP Flowcell in paired-end 2x100 mode. Demultiplexing was performed using bcl2fastq v2.20.0. Reads were aligned to GRCh38 and processed using the rna-seq-star-deseq2 snakemake-workflow version 1.1.2 (https://github.com/snakemake-workflows/rna-seq-star-deseq2; DOI: https://doi.org/10.5281/zenodo.4737358[8]) with default parameters. Differentially expressed genes were visualized by Prism 9.1.0. Gene set enrichment analysis (GSEA) was performed by use of GSEA 4.1.0 with a log2fold-change preranked list. The MSigDB curated C2 gene sets collection v7.4 (including BIOCHARTA, KEGG, PID, REACTOME and WIKIPATHWAYS) was used to identify enrichments.

Supplementary tables

Table S1: sgRNA sequences for CRISPR/Cas9 editing

Name	sequence (5’-3’)	PAM
ASXL1_ex13	GCCACCACTGCCATCGGAGG	GGG
DNMT3A_ex23_T1	CGTCTCCAACATGAGCCGCT	TGG
TET2_ex6	ACGGCACGCTCACCAATCGC	CGG

Ex: exon; PAM: Protospacer adjacent motif

Table S2: Antibodies for Dot Blot and Western Blot.

Antibody	Host	Conjugate	Clone	Company
5-Hydroxymethylcytosin (5-hmC) antibody	rabbit	-	polyclonal	Active Motif (39770)
5-Methylcytosin (5-mC)	rabbit	-	D3S2Z	Cell Signaling Technology (28692)
ASXL1 antibody middle region	rabbit	-		Aviva Systems Biology (OAAB07377)
DNMT3A antibody	mouse	-	C-12	Santa Cruz Biotechnology (sc-365769)
TET2 antibody	rabbit	-	polyclonal	Abcam (ab94580)
β-Actin antibody	mouse	-	C-4	Santa Cruz Biotechnology (sc-47778)
Goat Anti-Mouse Immunoglobulins	goat	HRP	polyclonal	Agilent Dako (P044701-2)
Swine Anti-Rabbit Immunoglobulins	swine	HRP	polyclonal	Agilent Dako (P021702-2)

Table S3: Antibodies for Flow Cytometry.

Antibody	Host	Conjugate	Clone	Company
anti-human CD14	mouse	APC-	61D3	eBioscience™ (17-0149)
anti-human CD14	mouse	Brilliant Violet 510™	M5E2	BioLegend® (301841)
anti-human CD19	mouse	PE-Cy™ 7	HIB19	BD Pharmingen™ (560728)
anti-human CD235a	mouse	FITC	HI264	BioLegend® (349104)
anti-human CD3	mouse	APC	HIT3a	BioLegend® (300311)
anti-human CD34	mouse	APC	581	BD Pharmingen™ (555824)
anti-human CD34	mouse	FITC	561	BioLegend® (343604)
anti-human CD34	mouse	PE	581	BD Phamingen™ (555822)
anti-human CD38	mouse	Alexa Fluor® 700	HIT2	BD Phamingen™ (560676)
anti-human CD38	mouse	PE/Cyanine 7	HIT2	BioLegend® (303516)
anti-human CD41	mouse	FITC	HIP8	BioLegend® (303704)
anti-human CD56	mouse	PE/Cyanine 7	5.1H11	BioLegend® (362510)
anti-human CD66b	mouse	Pacific Blue™	G10F5	BioLegend® (305112)
anti-human CD90	mouse	PerCP/Cyanine5.5	5E10	BioLegend® (328118)
anti-human FcεRIα	mouse	PE	AER-37	BioLegend® (334610)

Table S4: Differentially expressed genes and DE genes associated with hematopoietic lineage regulation[9-22] in *TET2* mutants.

Table is provided as separate spreadsheet file

Table S5: Variants identified by deep sequencing.

Table is provided as separate spreadsheet file

Supplementary figures

Figure S1: Medium composition for short-term culture. A) Flow cytometry analysis of unmodified cells in different culture media after 7, 14, and 21 days in culture: CD19 B cells, CD34 and $CD34^{+}CD38^{-}$ progenitor cells, CD66b granulocytes, CD41 megakaryocytes, and CD14 monocytes. The bars show mean and standard deviation of duplicates. B) Cytokine cocktails for $CD34^{+}$ cell culture. SCF: stem cell factor, FLT3-L: FMS-like tyrosine kinase 3 ligand, TPO: thrombopoietin, IL-3: interleukin 3, IL-6: interleukin 6, G-CSF: granulocyte-colony stimulating factor, GM-CSF: granulocyte macrophage-colony stimulating factor, EPO: erythropoietin.

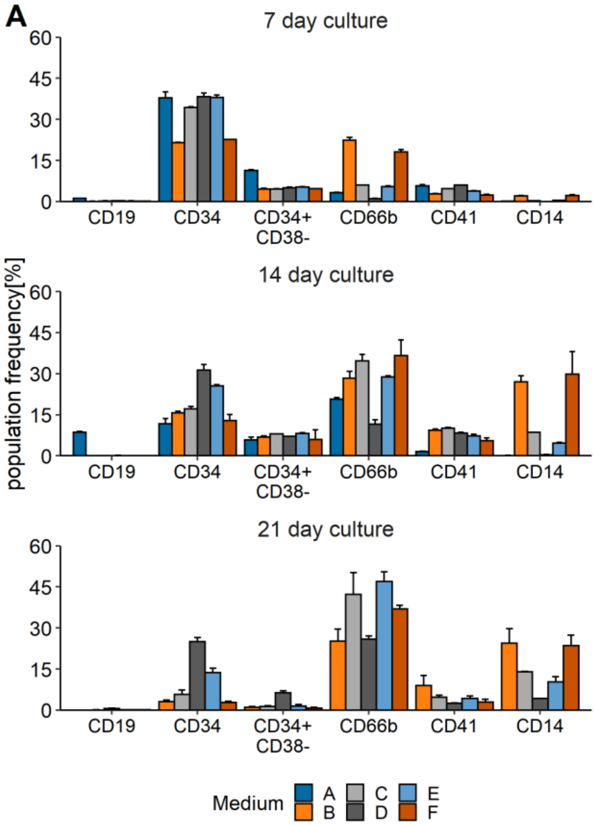

B

	A	B	C	D	E	F
**SCF**	0	100	100	50	100	25
**FLT3-L**	0	100	100	50	100	10
**IL3**	0	0	20	10	100	10
**IL6**	0	0	20	20	100	10
**TPO**	0	100	0	50	100	10
**G-CSF**	0	0	0	0	100	20
**GM-CSF**	0	0	0	0	100	5
**EPO**	0	0	0	0	0	10

Figure S2: Flow cytometry analysis of CD34^{+} enriched cells. Mononuclear cells were isolated from freshly collected umbilical cord blood and further enriched for CD34^{+} cells by magnetic separation (MACS). After 2 days of culture in expansion medium, the cells were analyzed via flow cytometry before transfection. CD14 monocytes, CD19 B cells, CD235a erythrocytes, CD3 T cells, CD34 progenitor cells, CD41 megakaryocytes, CD56 NK cells, CD66b granulocytes. The numbers above the bars indicate the frequency of the respective population in %.

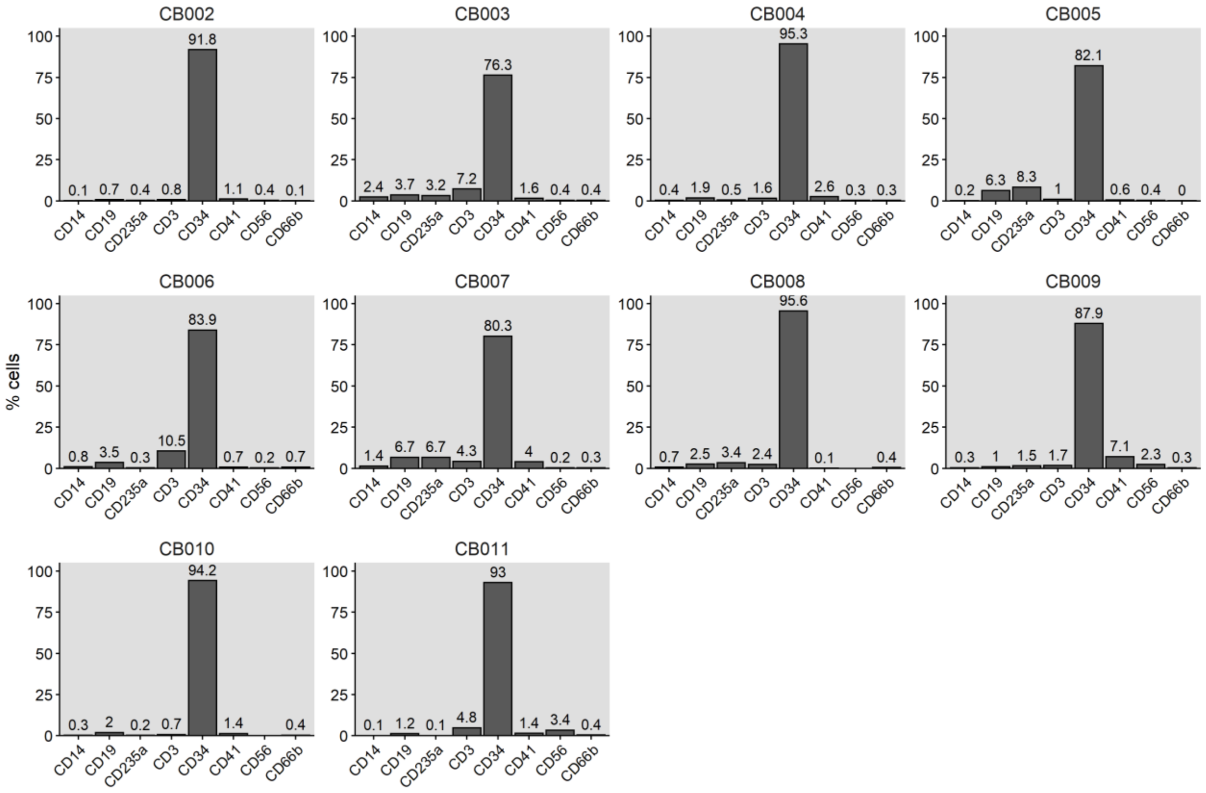

Figure S3: Target knockout in CD34⁺ cells. A + B) Quantitative real-time PCR analysis of *ASXL1*, *DNMT3A*, and *TET2* mRNA after CRISPR knockout of the respective targets. Relative expression was calculated via the $\Delta\Delta C_T$-method with the sample transfected only with Cas9 protein as reference sample (relative expression = 0). *GAPDH* was used as endogenous control. A) Replicates (three technical, two biological) of three specimens were analyzed after 3 days and of two specimens also 7 and 14 days after transfection. Bars and error bars show mean (SD). FDR-corrected multiple paired t-tests were conducted against wild type controls after testing for normal distribution. Decreased abundance of transcripts in the knockout samples could only be confirmed in early time points for the *TET2* variant B) Western Blot analysis of TET2, DNMT3A and ASXL1 protein expression of n≥3 biological samples after 21 days. The signal intensity was measured and normalized based on ponceau total protein stain. Relative intensity was calculated in respect to the WT sample. WT: Cells transfected with Cas9 protein. *DNMT3A* and *ASXL1* variants slowed a slight decrease in protein expression, whereas the TET2 protein level was highly variable throughout the replicates after 21 days.

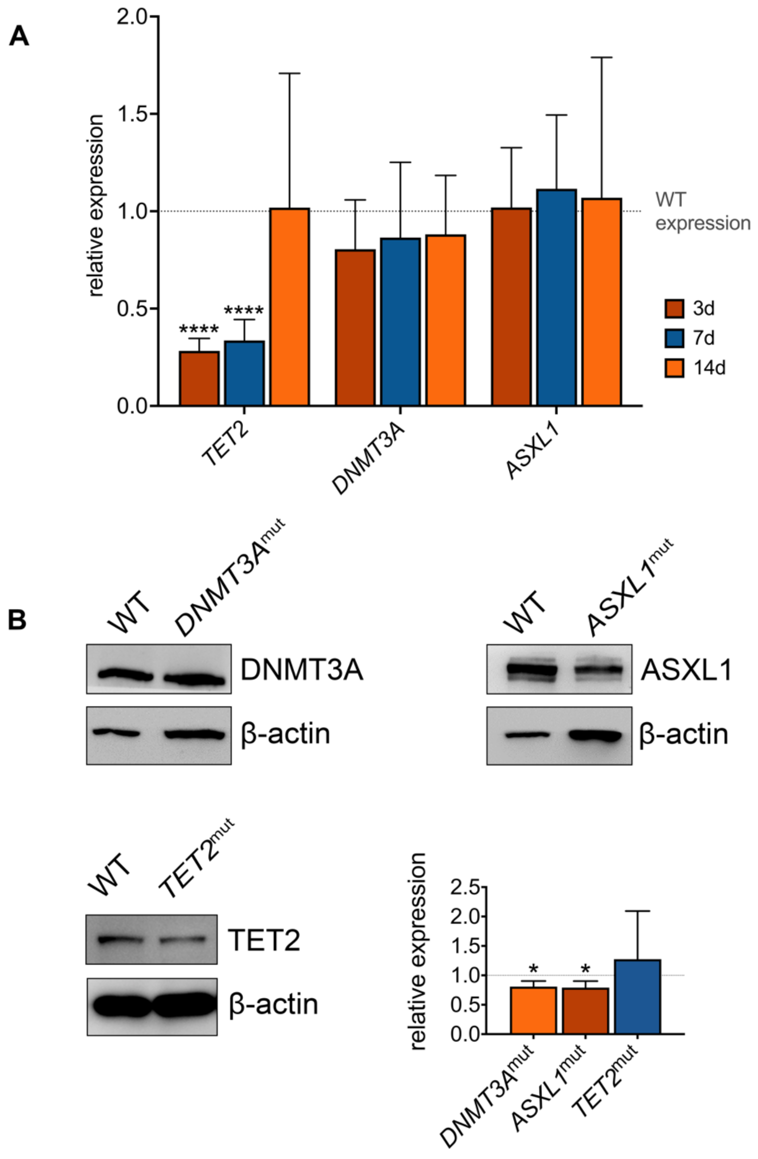

Figure S4: Dot Blot analysis of 5-hydroxymethylcytosin and 5-methylcytosin levels. A) Relative 5hmC levels of *DNMT3A*^{mut} and *TET*2^{mut} cells compared to wild type cells (Cas9 protein only). B) Relative 5mC levels of *DNMT3A*^{mut} and *TET*2^{mut} cells compared to wild type cells (Cas9 protein only). Signal intensities were quantified via ImageJ, normalized to the methylene blue loading control, and calculated relative to the wild type sample for each time point and input amount of three biological samples. The bars show mean and standard deviation of the relative signal intensity (n ≥4), Kruskal-Wallis test with Dunn's multiple comparisons test demonstrated no significant differences except from 5hmC level in *TET2* altered samples versus WT after 19 days of culture. C) One representative example of three independent 5hmC and 5mC dot blots. Different time points are indicated above the blot (d = days after transfection), and different input amounts are shown on the left. Detections of 300 ng and 150 ng were carried out in one single blot per biological sample, but figure was cut and compressed to increase conciseness.

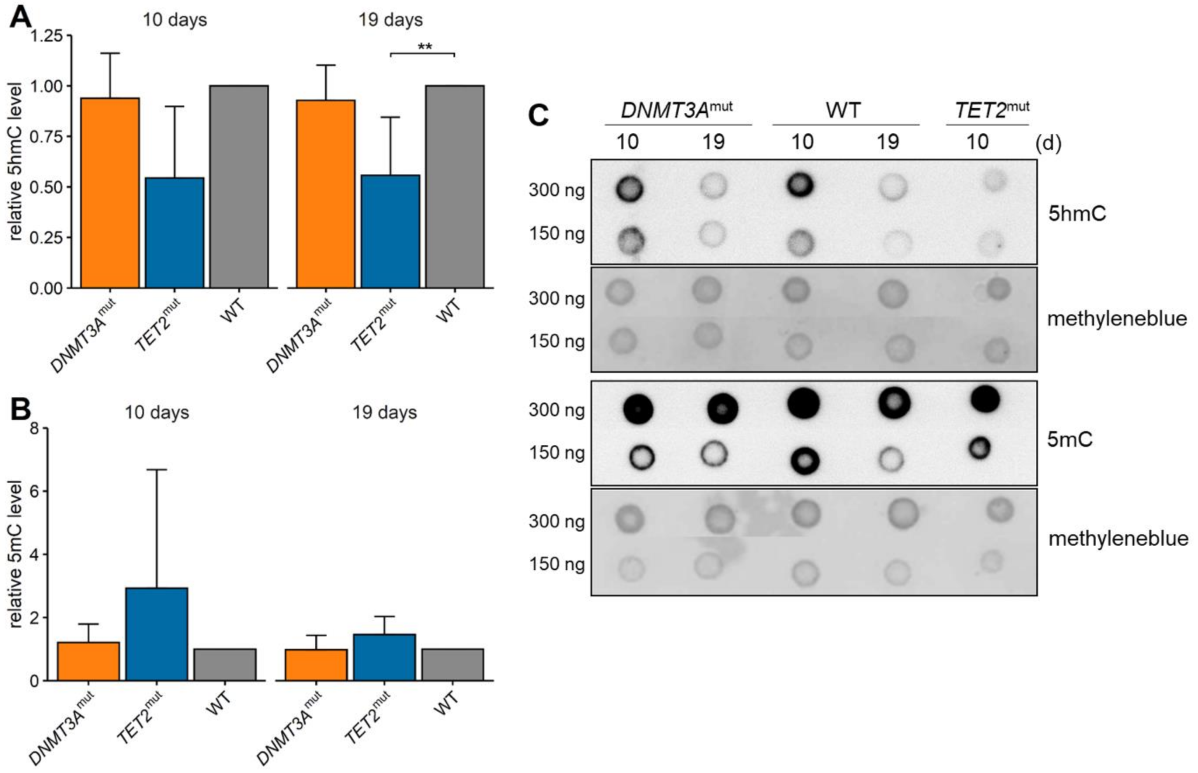

Figure S5: Cell surface marker analysis via flow cytometry of A) *TET2*^{mut} and multi KO vs WT and B) *DNMT3A*^{mut}, *ASXL1*^{mut} and *TET2*^{mut} vs WT. Cells were analyzed 7, 14, and 21 days post transfection. CD19 B cells, CD235a erythroid cells, CD34 progenitor cells, CD66b granulocytes, FcεRIα mast cells and basophiles, CD41 megakaryocytes, CD14 monocytes, CD3 T cells. Frequencies were normalized to 100% for all depicted cell populations. Bars and error bars show mean and standard deviation of two transfection replicates. No significant changes were determined.

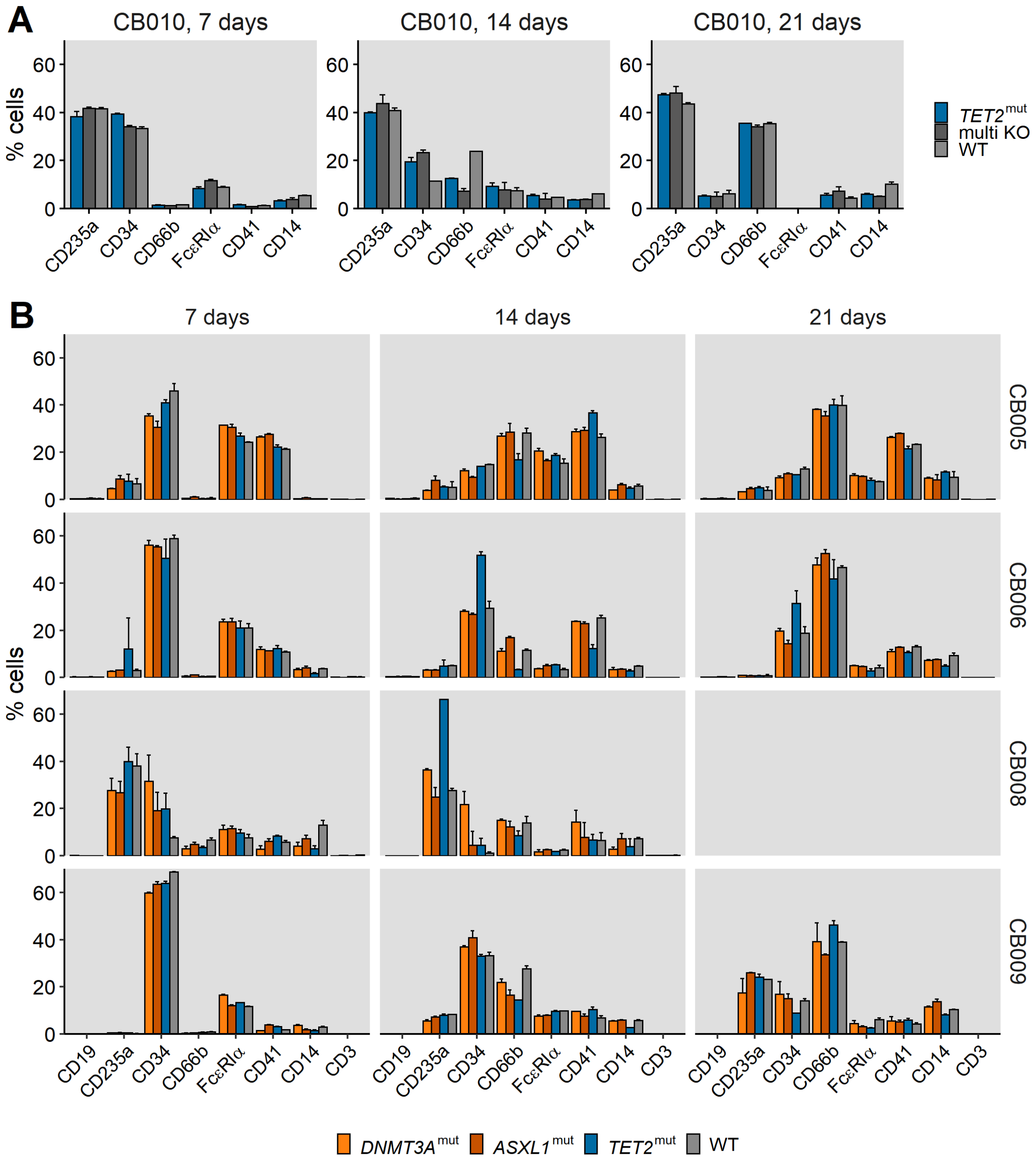

Figure S6: Colony forming unit and serial replating assays of *TET2*^{mut} CD34^{+} cells. Bar plots show the raw data of different biological samples. Colonies were determined as BFU.E: burst forming unit erythroid, CFU.E: colony forming unit erythroid, CFU.GM: colony forming unit granulocyte, macrophage, CFU.GEMM: colony forming unit granulocyte, erythroid, macrophage, megakaryocyte per 1,000 plated cells. Biological replicates are indicated above the bars. The number of the passage is shown on the right (p1: first seeding, p2: second seeding, p3: third seeding). All bars show mean and standard deviation of two technical replicates.

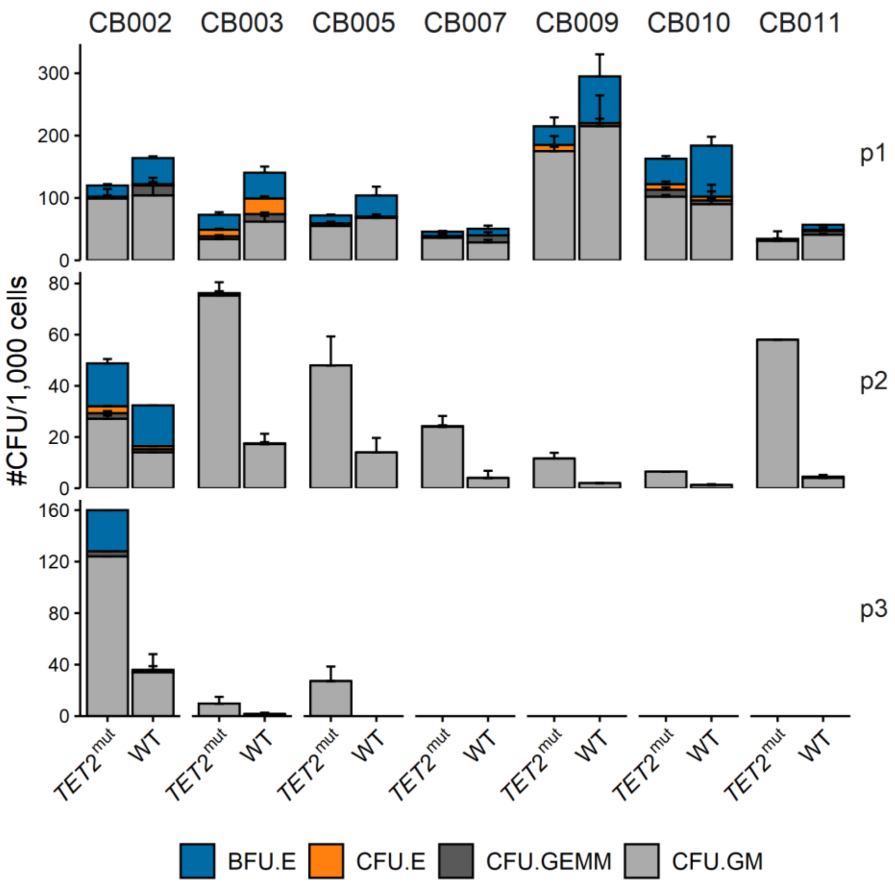

Figure S7: Serial replating of A) *DNMT3A*mut and *ASXL1*mut vs. WT cells of four biological replicates and B) multi KO vs. WT cells of three biological replicates. The number of passages is indicated above the plots. No significant differences were obtained, Wilcoxon rank-sum test.

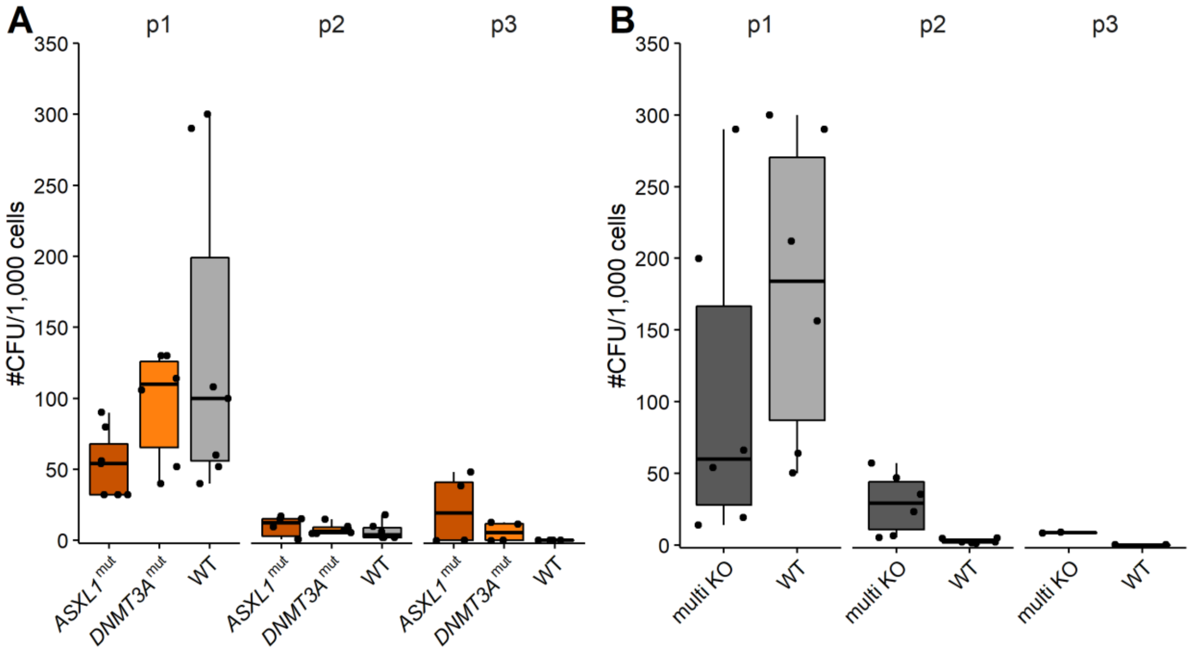

Figure S8: Pathway analysis of DE genes in *TET2*mut cells. Differentially expressed genes 7, 14, and 21 days after knockout of *TET2* were analyzed by Gene Set Enrichments Analyses (GSEA) using the curated C2 collection from the Molecular Signature Database (MSigDB; https://www.gsea-msigdb.org[23]). A) VENN diagram shows significantly enriched gene sets 7,14 and 21 days after transfection (Table S4). B) Four gene sets were enriched at all three time points: KEGG_ANTIGEN_PROCESSING_AND_PRESENTATION, KEGG_ASTHMA, KEGG_HEMATOPOIETIC_CELL_LINEAGE, REACTOME_IMMUNOREGULATORY_INTERACTIONS_BETWEEN_A_LYMPHOID_AND_A_NON_LYMPHOID_CELL (R-HSA-198933), all of which are associated with immune cell communication, antigen presentation and differentiation. Most notably, we found a strong enrichment of the hematopoietic cell lineage gene set in all our analyses. Enrichments blots are shown. C) KEGG mapping of early significantly altered genes 7 days after transfection to the KEGG hematopoietic cell lineage gene set demonstrated a predominant suppression of genes associated with myeloid lineage features related to wild type.

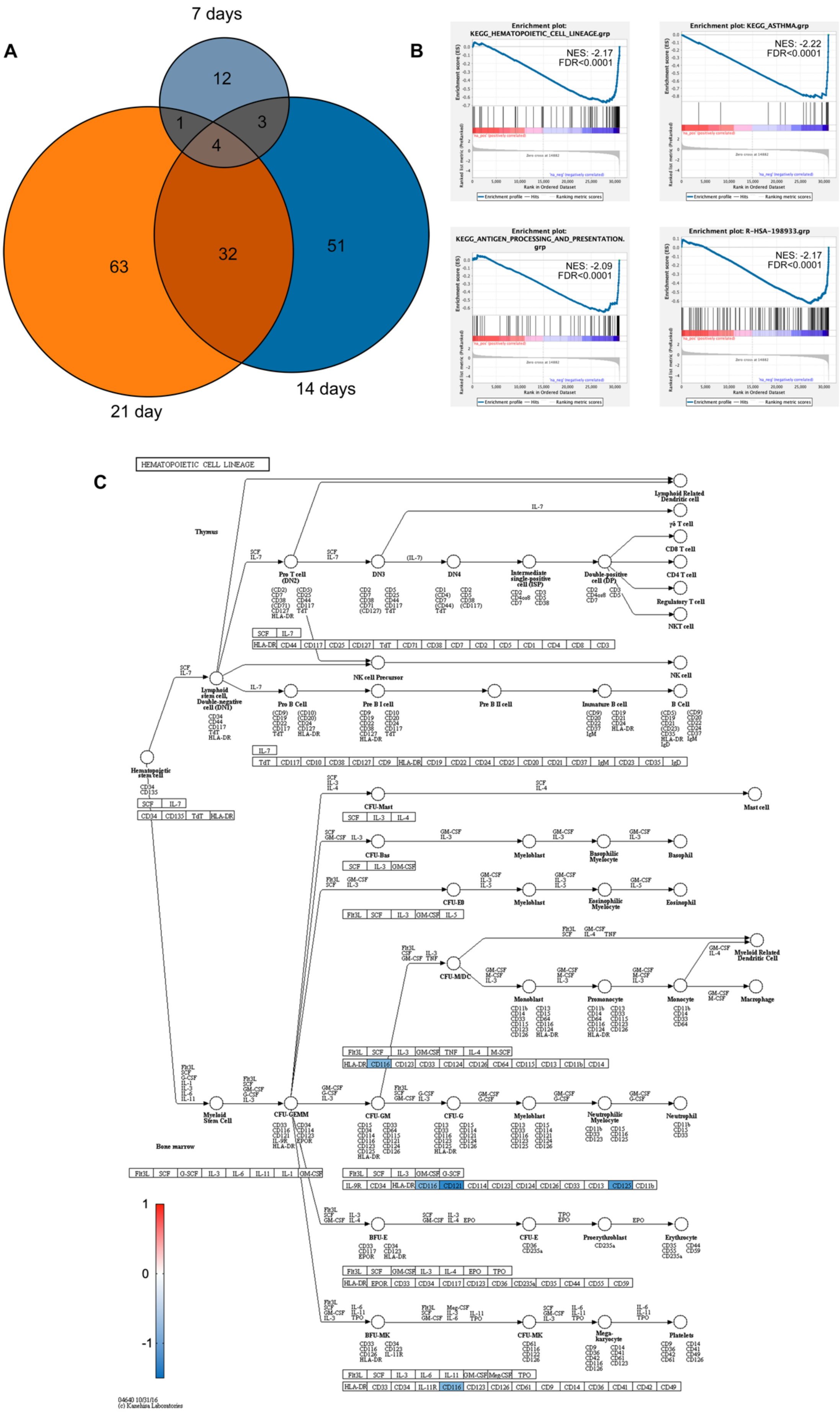
A
7 days
12
1
3
4
63
32
51
14 days
21 day
B
Enrichment plot: KEGG_HEMATOPOIETIC_CELL_LINEAGE.grp
NES: -2.17
FDR<0.0001
Enrichment plot: KEGG_ASTHMA.grp
NES: -2.22
FDR<0.0001
Enrichment plot: KEGG_ANTIGEN_PROCESSING_AND_PRESENTATION.grp
NES: -2.09
FDR<0.0001
Enrichment plot: R-HSA-198933.grp
NES: -2.17
FDR<0.0001
C
HEMATOPOIETIC CELL LINEAGE
Thymus
Bone marrow
Hematopoietic stem cell
Lymphoid stem cell, Double-negative cell (DN1)
Pro T cell (DN2)
DN3
DN4
Intermediate single-positive cell (ISP)
Double-positive cell (DP)
Lymphoid Related Dendritic cell
γδ T cell
CD8 T cell
CD4 T cell
Regulatory T cell
NKT cell
NK cell Precursor
NK cell
Pro B Cell
Pre B I cell
Pre B II cell
Immature B cell
B Cell
Myeloid Stem Cell
CFU-GEMM
CFU-Mast
Mast cell
CFU-Bas
Myeloblast
Basophilic Myelocyte
Basophil
CFU-EB
Eosinophilic Myelocyte
Eosinophil
CFU-M/DC
Myeloid Related Dendritic Cell
Monoblast
Promonocyte
Monocyte
Macrophage
CFU-GM
CFU-G
Neutrophilic Myelocyte
Neutrophil
BFU-E
CFU-E
Proerythroblast
Erythrocyte
BFU-MK
CFU-MK
Mega-karyocyte
Platelets
1
0
-1

Figure S9: Predicted indel frequencies for different time points and culture conditions as predicted by CRISPRseq analysis. A) Indel frequencies of short-term culture samples. The target is indicated above the plot and the time point is shown on the x-axis. B) Indel frequencies compared between culture conditions for each target. No significant differences were determined by Wilcoxon rank-sum test, p-values were corrected for multiple testing.

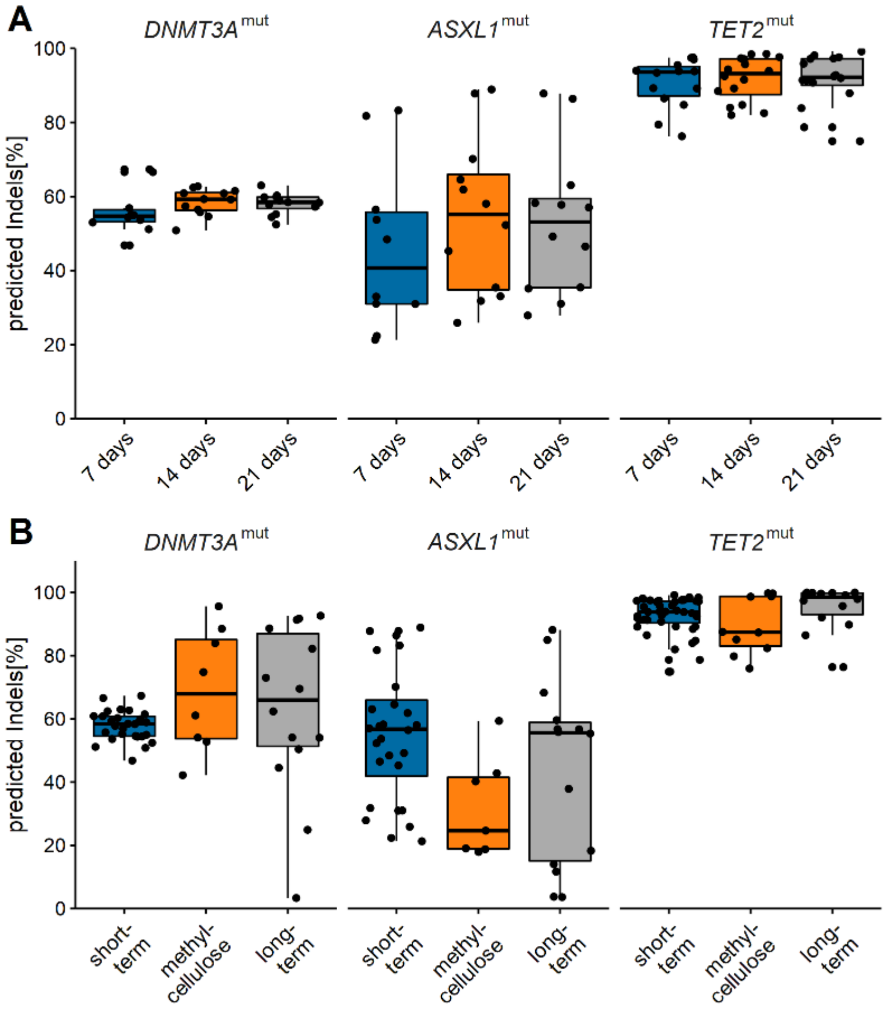

Figure S10: Effect of non-randomly induced indels on clonal selection. Rational indel meta-analysis (RIMA) was performed to quantify the mean variant allele frequency (VAF) of c-MMEJ-associated variants 7 days after genome editing and after long-term culture in n=4 biological replicates. A) Mean (SD) VAF of c-MMEJ-derived indels before and after long-term culture for each target gene and overall showed a slightly, but not significantly higher increase in VAF in c-MMEJ versus non-c-MMEJ derived variant. B) Relative changes in mean (SD) VAF after long-term culture demonstrated that there was no higher selective advantage of non-randomly induced indels compared to other variants (one-way ANOVA corrected for multiple testing).

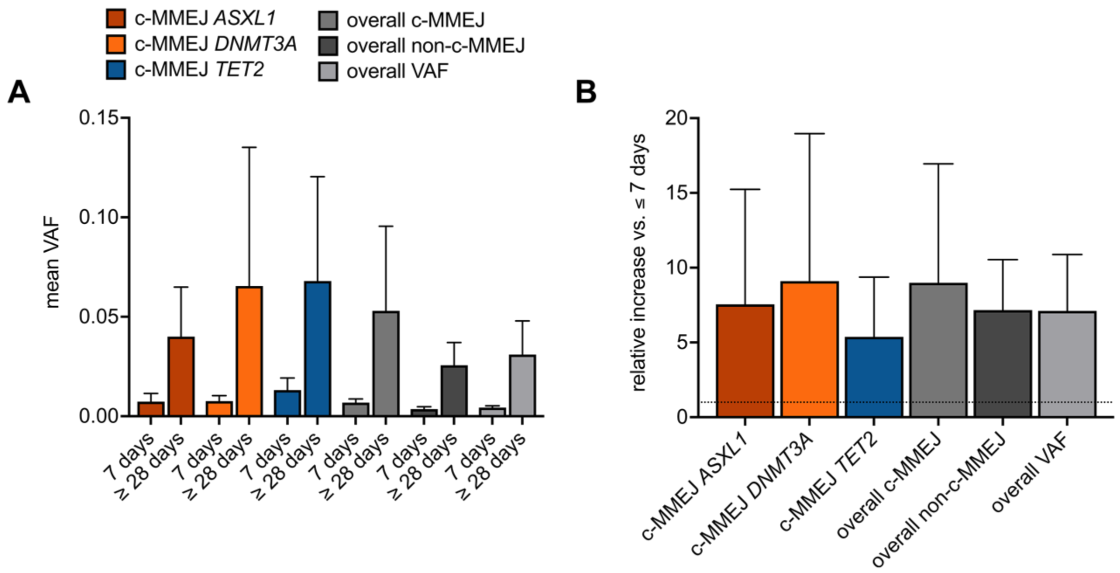

Figure S11: Mutational composition of *ASXL1*^{mut} samples. A) Short-term culture (7 days) and B) long-term culture. The biological replicate is indicated above the plots. The annotation above the bars shows the overall indel frequency for the short-term culture samples. The annotation on the bars indicates the protein consequence of the respective mutation. The 30 most frequent mutations are shown and the annotated variants are highlighted in the legend.

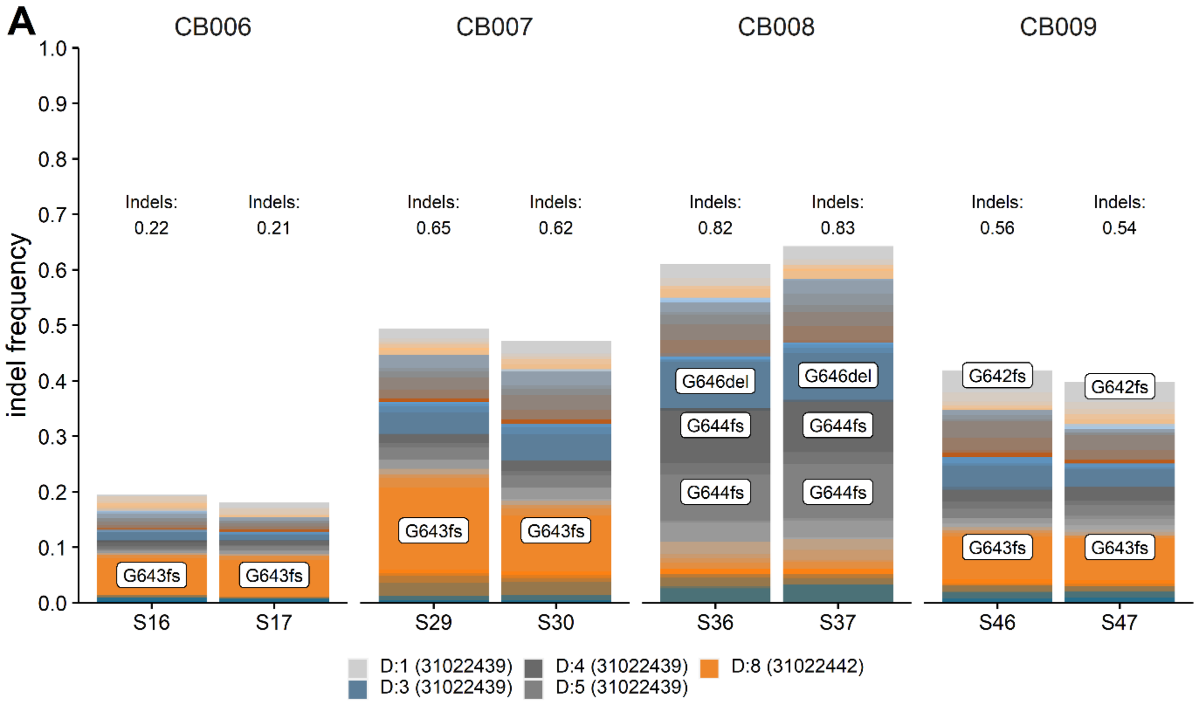

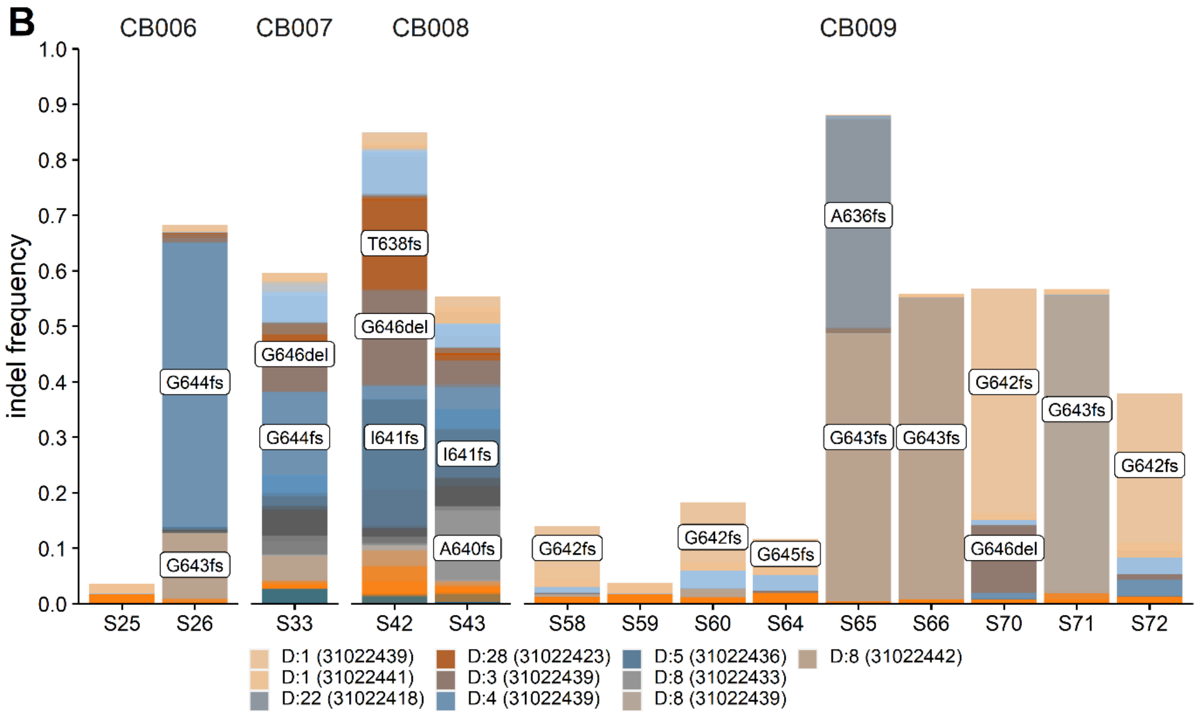

Figure S12: Mutational composition of *DNMT3A*^{mut} samples. A) Short-term culture (7 days) and B) long-term culture. The biological replicate is indicated above the plots. The annotation above the bars shows the overall indel frequency as well as the frequency of the point mutation (SNV) for the short-term culture samples. The annotation on the bars indicates the protein consequence of the respective mutation. The 30 most frequent mutations are shown and the annotated variants are highlighted in the legend.

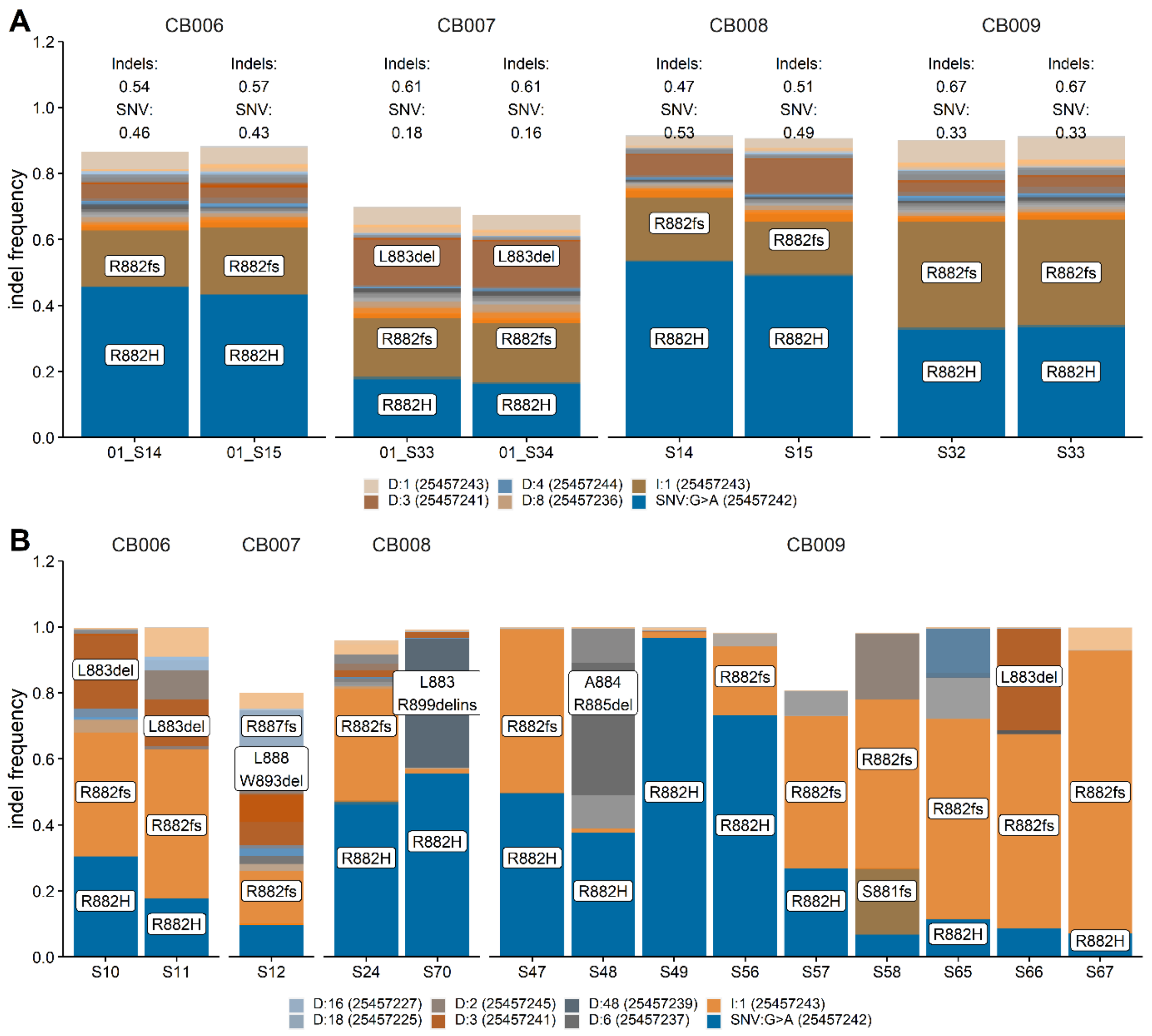

Figure S13: Mutational composition of *TET2*^{mut} samples. A) Short-term culture (7 days) and B) long-term culture. The biological replicate is indicated above the plots. The annotation above the bars shows the overall indel frequency for the short-term culture samples. The annotation on the bars indicates the protein consequence of the respective mutation. The 30 most frequent mutations are shown and the annotated variants are highlighted in the legend.

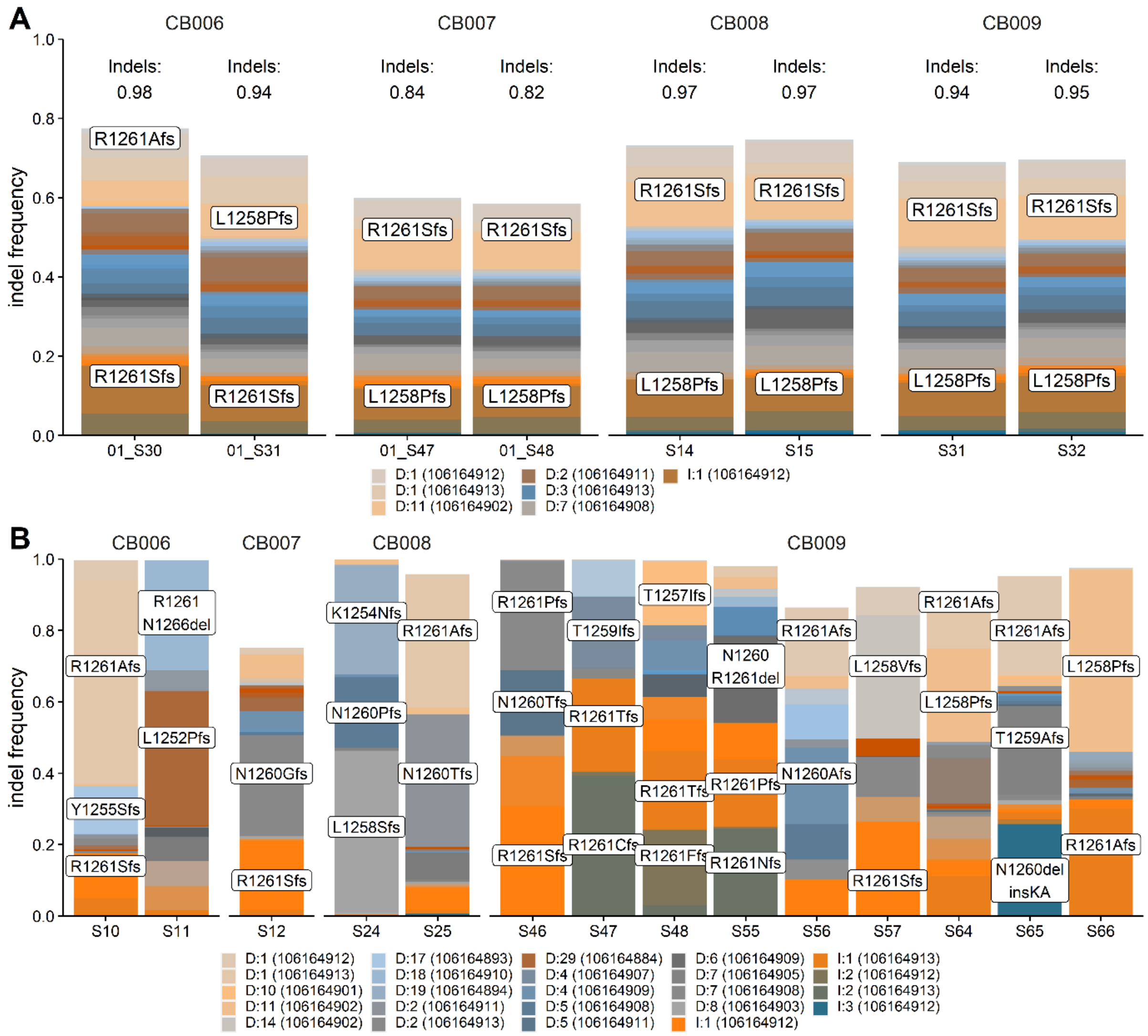

Supplementary References

1. Brunetti L, Gundry MC, Kitano A, Nakada D, Goodell MA. Highly Efficient Gene Disruption of Murine and Human Hematopoietic Progenitor Cells by CRISPR/Cas9. *Journal of visualized experiments : JoVE* 2018; (134).

2. Hsiau T, Conant D, Rossi N, Maures T, Waite K, Yang J*, et al. Inference of CRISPR Edits from Sanger Trace Data*, 2018.

3. Ncbi.nlm.nih.gov. GRCh37 - hg19 - Genome - Assembly - NCBI. [cited 06.12.2020]; Available from: https://www.ncbi.nlm.nih.gov/assembly/GCF_000001405.13/

4. Li H. Aligning sequence reads, clone sequences and assembly contigs with BWA-MEM. 2013. p. 3.

5. Tothova Z, Krill-Burger JM, Popova KD, Landers CC, Sievers QL, Yudovich D*, et al.* Multiplex CRISPR/Cas9-Based Genome Editing in Human Hematopoietic Stem Cells Models Clonal Hematopoiesis and Myeloid Neoplasia. *Cell stem cell* 2017; **21**(4)**:** 547-555.e548.

6. Poplin R, Chang P-C, Alexander D, Schwartz S, Colthurst T, Ku A*, et al.* A universal SNP and small-indel variant caller using deep neural networks. *Nature biotechnology* 2018; **36**(10)**:** 983–987.

7. Taheri-Ghahfarokhi A, Taylor BJ M, Nitsch R, Lundin A, Cavallo A-L, Madeyski-Bengtson K*, et al.* Decoding non-random mutational signatures at Cas9 targeted sites. *Nucleic Acids Research* 2018; **46**(16)**:** 8417-8434.

8. Köster J, Forster J, Schmeier S, Parsons L. Snakemake workflow: rna-seq-star-deseq2. 2017.

9. Doulatov S, Notta F, Laurenti E, Dick John E. Hematopoiesis: A Human Perspective. *Cell Stem Cell* 2012 2012/02/03/; **10**(2)**:** 120-136.

10. Buenrostro JD, Corces MR, Lareau CA, Wu B, Schep AN, Aryee MJ*, et al.* Integrated Single-Cell Analysis Maps the Continuous Regulatory Landscape of Human Hematopoietic Differentiation. *Cell* 2018 May 31; **173**(6)**:** 1535-1548.e1516.

11. Orkin SH, Zon LI. Hematopoiesis: An Evolving Paradigm for Stem Cell Biology. *Cell* 2008 2008/02/22/; **132**(4)**:** 631-644.

12. Shivdasani RA, Orkin SH. The transcriptional control of hematopoiesis. *Blood* 1996 May 15; **87**(10)**:** 4025-4039.

13. Laiosa CV, Stadtfeld M, Graf T. Determinants of lymphoid-myeloid lineage diversification. *Annu Rev Immunol* 2006; **24:** 705-738.

14. Lulli V, Romania P, Morsilli O, Gabbianelli M, Pagliuca A, Mazzeo S*, et al.* Overexpression of Ets-1 in human hematopoietic progenitor cells blocks erythroid and promotes megakaryocytic differentiation. *Cell Death & Differentiation* 2006 2006/07/01; **13**(7)**:** 1064-1074.

15. Fleury M, Eliades A, Carlsson P, Lacaud G, Kouskoff V. FOXF1 inhibits hematopoietic lineage commitment during early mesoderm specification. *Development* 2015 Oct 1; **142**(19)**:** 3307-3320.

16. Burda P, Laslo P, Stopka T. The role of PU.1 and GATA-1 transcription factors during normal and leukemogenic hematopoiesis. *Leukemia* 2010 2010/07/01; **24**(7)**:** 1249-1257.

17. Balogh P, Adelman ER, Pluvinage JV, Capaldo BJ, Freeman KC, Singh S*, et al.* RUNX3 levels in human hematopoietic progenitors are regulated by aging and dictate erythroid-myeloid balance. *Haematologica* 2020 Apr; **105**(4)**:** 905-913.

18. Perry SS, Zhao Y, Nie L, Cochrane SW, Huang Z, Sun X-H. Id1, but not Id3, directs long-term repopulating hematopoietic stem-cell maintenance. *Blood* 2007; **110**(7)**:** 2351-2360.

19. Wang H, Wang M, Wang Y, Wen Y, Chen X, Wu D*, et al.* MSX2 suppression through inhibition of TGFβ signaling enhances hematopoietic differentiation of human embryonic stem cells. *Stem Cell Research & Therapy* 2020 2020/04/05; **11**(1)**:** 147.

20. Pajcini KV, Speck NA, Pear WS. Notch signaling in mammalian hematopoietic stem cells. *Leukemia* 2011 2011/10/01; **25**(10)**:** 1525-1532.

21. Huang X, Lee MR, Cooper S, Hangoc G, Hong KS, Chung HM*, et al.* Activation of OCT4 enhances ex vivo expansion of human cord blood hematopoietic stem and progenitor cells by regulating HOXB4 expression. *Leukemia* 2016 2016/01/01; **30**(1)**:** 144-153.

22. Kuo YH, Zaidi SK, Gornostaeva S, Komori T, Stein GS, Castilla LH. Runx2 induces acute myeloid leukemia in cooperation with Cbfbeta-SMMHC in mice. *Blood* 2009 Apr 2; **113**(14)**:** 3323-3332.

23. Subramanian A, Tamayo P, Mootha VK, Mukherjee S, Ebert BL, Gillette MA*, et al.* Gene set enrichment analysis: a knowledge-based approach for interpreting genome-wide expression profiles. *Proceedings of the National Academy of Sciences of the United States of America* 2005 Oct 25; **102**(43)**:** 15545-15550.
